# Oxygen-Driven Tumour Growth Model: A Pathology-Relevant Mathematical Approach

**DOI:** 10.1371/journal.pcbi.1004550

**Published:** 2015-10-30

**Authors:** Juan A. Delgado-SanMartin, Jennifer I. Hare, Alessandro P. S. de Moura, James W. T. Yates

**Affiliations:** 1 Modelling & Simulation Oncology DMPK, AstraZeneca, Cambridge, United Kingdom; 2 Physics Department, University of Aberdeen, Aberdeen, United Kingdom; 3 *In vivo* bioscience, AstraZeneca, Macclesfield, United Kingdom; National Research Council of Canada, CANADA

## Abstract

Xenografts -as simplified animal models of cancer- differ substantially in vasculature and stromal architecture when compared to clinical tumours. This makes mathematical model-based predictions of clinical outcome challenging. Our objective is to further understand differences in tumour progression and physiology between animal models and the clinic.

To achieve that, we propose a mathematical model based upon tumour pathophysiology, where oxygen -as a surrogate for endocrine delivery- is our main focus. The Oxygen-Driven Model (ODM), using oxygen diffusion equations, describes tumour growth, hypoxia and necrosis. The ODM describes two key physiological parameters. Apparent oxygen uptake rate ($k_{R}^{\prime }$) represents the amount of oxygen cells seem to need to proliferate. The more oxygen they appear to need, the more the oxygen transport. $k_{R}^{\prime }$ gathers variability from the vasculature, stroma and tumour morphology. Proliferating rate (*k*_*p*_) deals with cell line specific factors to promote growth. The *K*_*H*_,*K*_*N*_ describe the switch of hypoxia and necrosis. Retrospectively, using archived data, we looked at longitudinal tumour volume datasets for 38 xenografted cell lines and 5 patient-derived xenograft-like models.

Exploration of the parameter space allows us to distinguish 2 groups of parameters. ***Group 1*** of cell lines shows a spread in values of $k_{R}^{\prime }$ and lower *k*_*p*_, indicating that tumours are poorly perfused and slow growing. ***Group 2*** share the value of the oxygen uptake rate ($k_{R}^{\prime }$) and vary greatly in *k*_*p*_, which we interpret as having similar oxygen transport, but more tumour intrinsic variability in growth.

However, the ODM has some limitations when tested in explant-like animal models, whose complex tumour-stromal morphology may not be captured in the current version of the model. Incorporation of stroma in the ODM will help explain these discrepancies. We have provided an example. The ODM is a very simple -and versatile- model suitable for the design of preclinical experiments, which can be modified and enhanced whilst maintaining confidence in its predictions.

## Author Summary

Tumour-bearing animal models of cancer are needed to discover new drugs to treat cancer. We aim in this article to capture—through mathematics- some underlying phenomena of tumour growth in animals. We propose a set of equations that, despite being very simple, describe tumour growth, hypoxia and necrosis. Cells under low oxygen levels change into a stress state called “hypoxia”, which will ultimately lead to tissue death, also known as “necrosis” and “apoptosis”. Hypoxic cells undergo a variety of changes which impact tumour growth, development, metastasis and -most importantly- response to therapy. Hence, oxygen distribution is important. We simulate oxygen profiles to locate hypoxic and necrotic tumour regions. Finally, this mathematical model allows us to compare and classify animal models from a grounded and physiological perspective, rather than a more convenient and empirical one. This will help us understand how well (or poorly) animal tumours mimic tumours in patients. The simplicity of our mathematical model allows us to obtain more information out of the same animal sets without any further experiments, hopefully saving time, money and animal usage.

## Introduction

Mathematical modelling of biological systems is a powerful tool to contrast hypotheses, thus enhancing our ways to interpret the often very complex data generated in drug discovery. In cancer, mathematical modelling has been widely applied to describe both clinical and *in vivo* preclinical tumours [1–3], however translation of animal data into the clinic remains still as a challenging task [4].

Mechanistic mathematical models of cancer endeavour to understand biological phenomena beyond its mere description. These models are based upon the gathering of cross-disciplinary knowledge of the biological system in question. For example, to understand vessel perfusion, you may need histological data, blood flow data and even evidence of the tumour mechanics, plus a collection of assumptions [5,6]. Some of these models also include simple drug effects [7] and more complex transient-stage drug models [8]. Also, some recent *in silico* models have been applied to clinical data [9], though being often insufficiently translatable across preclinical and clinical settings. Agent-based *in silico* models of tumour growth and proliferation of vessels and lymph are useful to identify vessel architectures and their mechanisms of progression [10,11], but, hitherto their validation using experiments are difficult.

The tumour microenvironment differs substantially between animal models and the clinic. This has been shown to be a key factor for the lack of success in clinical outcome prediction [12]. More specifically tumour-stroma interaction is said to be the main cause for tumour progression and metastasis [12]. Further, some *in silico* modelling techniques, based on agent-based and game theory between stromal-tumour cell populations have recently been discussed and contrasted *in vitro* [13], although again with no insight towards animal-clinical translation.

### Tumour pathophysiology

We hypothesise connecting tumour-stromal morphology to growth is an important step to enable a description of the differences in preclinical and clinical tumours. In this first step, we consider an avascular tumour analogous to a tumour nest surrounded by stroma, which is a common morphology found in xenografted tumours. We hypothesise, that stromal and vascular phenotypes and morphologies influence delivery of nutrient and oxygen, and thereby tumour growth [14–15].

Many novel therapeutic approaches endeavour to tackle cancer by systemic exposure to agents (~chemotherapy). These approaches include encapsulation of small molecules (nanomedicine), biologics, personalised therapy, immune therapy, antiangiogenics and many more. Clinical outcome predicted from some of these therapeutic approaches tested in animals is especially poor, mostly due to the poor understanding of differences in tumour pathophysiology; e.g. antiangiogenic and nanomedicines [16–18]. Today only a handful of these drugs are in the clinic [18], despite the very promising results observed in animals.

### The tumour growth Oxygen-Driven Model (ODM)

Oxygen is an important molecule, whose uptake, utilisation, diffusion and metabolism in cells have been thoroughly studied from a biological point of view [19–22]. A number of researchers have simulated oxygen profiles along with other key aspects of tumour progression, like microenvironment and biomechanics [23], also in a multiscalar manner [11]. Some of these mathematical models have been validated *in vivo* [24]. However, this validation is demanding and the results are unsuitable to be applied extensively in a drug discovery setting.

We aim for an *in silico* tumour growth model which describes aspects of the tumour pathophysiology, with an emphasis on oxygen delivery as a surrogate for other molecules coming from the vasculature. The mathematical model should contain information on hypoxic and necrotic regions. Nonetheless, the key aspect is the comparability across animal models and to the clinic. Its simplicity allows a concise estimation of the parameters with small data sets and will be the first step to a deeper understanding of how stroma, vasculature and tumour interact in preclinical biological models.

### ODM development

The starting point of this *in silico* model stems from previous radial models [25], combined with observations and models made on the relationship between oxygen and proliferation rates [23,26,27]. *In vitro* doubling times are substantially shorter than *in vivo* [28] and clinical [29] doubling times, which can be attributed to restricted delivery of nutrient and oxygen *in vivo*. We also believe mathematical models do not fully exploit the potential of the data, by being overly complex and having unidentifiable parameters [27]. In this sense, we developed an *in silico* model evolving from large models of hypoxia, using geometric features of tumours and simple physical equations. More information on the development of the ODM and some further investigations are explained in detail in the S1 Text.

## Results

### The ODM’s Mathematical Formulation

**Table 1:** Variable and parameter glossary.

Symbol	Name	Units	Description
*V*_*i*_	Tumour volume	cm^3^	Tumour volume of each tumour layer
$V_{i}^{H}$	Hypoxic volume	cm^3^	Volume of tumour layer being hypoxic
$V_{i}^{N}$	Necrotic volume	cm^3^	Volume of tumour layer being necrotic
*V*_*T*_	Tumour volume	cm^3^	Total tumour volume (*V*_*T*_ = Σ_*i*_ *V*_*i*_)
*V*_*To*_	Initial tumour volume	cm^3^	Total tumour volume at experiment start
*r*_*T*_	Proportional to Tumour radius	cm	$r_{T}=V_{T}{}^{1/3}$
i	Layer #	-	
n	# of Layers	-	
$P_{O_{2},i}$	Oxygen Pressure	mmHg	Oxygen levels in each layer
$P_{O_{2}}{}^{\text{max}}$	Blood Oxygen Levels	mmHg	Maximum blood oxygen levels at tumour periphery
t	Time	day	Time elapsed after beginning of experiment
*k*_*p*_	Proliferation rate	(day mmHg)^-1^	Rate at which cells divide. Cell-type dependent
$k_{R}\prime$	Apparent oxygen uptake rate	cm^-1^	Apparent oxygen needed for cells to divide: kR′=kR/D⋅(34π)1/3; because this expression always appears as a product with *r*_*T*_, we include the (34π)1/3 to reduce computational burden and the presentation of the equations
*k*_*R*_	Oxygen uptake rate	day/cm^3^	Oxygen needed for cells to divide
*D*	Diffusion coefficient	cm^2^/day	Ease of oxygen to diffuse
*K*_*H*_	Hypoxia constant	mmHg	Sharpness of hypoxic switch
*K*_*N*_	Necrosis constant	mmHg	Sharpness of necrotic switch

### *In silico* model assumptions

We considered some common assumptions as a starting point, based on the literature:

- Tumour shape: spherical. In order to simplify the spatial discretisation the tumour is taken as one dimensional, where r, the radius is the only spatial variable [7,25].
- O_2_ transport occurs by molecular random walk monotony, i.e. diffusionally. There is no convection (flow is nearly stationary Re < 1) or active transport (no intermembrane active oxygen transporters). Hereby Fick’s law of molecular transport is applicable [30].
- Time scale: O_2_ diffusion (~1min^-1^) occurs on a faster timescale than cell division (~1day^-1^), where oxygen has reached steady state between each cell division.[31].
- Homogeneous tissue: constant diffusion (Fick’s diffusion constant remains 1.9x10^-6^cm^2^/s, as for (H^+^) [32], see also epithelial transmissibility of Oxygen 5.3x10^-11^(cm^2^.ml_O2_)/(s ml mmHg) [33] and 3x10^-10^(cm^2^ ml_O2_)/(s ml mmHg)[34]). This assumption has been implemented in many mathematical models including [35].
- Extracelullar matrix (ECM) is the paracellular connective tissue assumed to have oxygen partial pressure of breast cancerous epithelial tissue, i.e. 60mmHg [36]. This parameter is a mere orientation which implies a large variability between subjects and tissue origin, varying from 30 to 104 mmHg for functional epithelium [37].
- Blood flow accessibility occurs from the whole surrounding matrix of the tumour, from the subcutaneous side of a xenograft and from the underlying hypervascularised adipose tissue.
- Proliferation correlates with oxygen levels, given that the efficiency of aerobic energy production is 36 to 2 compared to the anaerobic glycolysis, even though there is a large upregulated transport of glucose towards cytosol [38].

### Mathematical formulation

**Fig 1:**
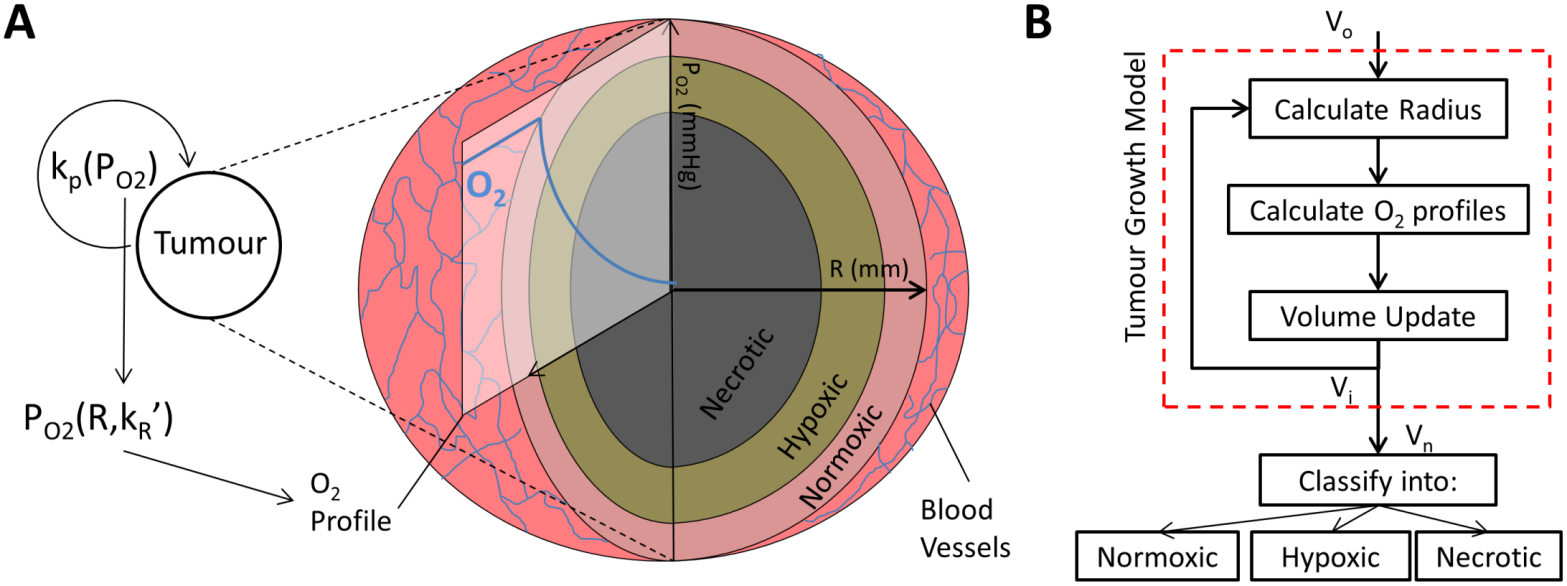
The ODM. (A) ODM sketch. (B) ODM iteration block diagram. In each iteration, we calculate radius, oxygen profile and update the volume. Hypoxia and necrosis are then calculated heuristically as proportions of the total volume.

As highlighted above, the ODM aims to describe the growth of xenografted tumours based upon their pathophysiology. The structure of the model will be described in depth hereafter, for which a graphical display is presented (see Fig 1A). Likewise, a sketch of the different steps of the iterative process is shown in Fig 1B. The S1 Text gives a historical perspective of the model and its evolution from previous models.

The ODM is composed of one non-linear differential equation (replicated in *n* number of concentric shells) –to describe tumour growth- and 2 algebraic equations –to describe the partitions of hypoxia and necrosis. The main assumption of the ODM is that **oxygen drives growth** in a proportional manner (see Eq (11)). Unlike for somatic tissue, tumours also create their own growth factors and cytokines locally [39]. However, tumour progression is associated with aerobic glycolysis [39,40], where blood is the only route of acquisition of oxygen. For that reason, the ODM is oxygen-centric.

The main equation relies on the physics of oxygen diffusion in porous media, for which first Fick’s and mass conservation laws were applied. Considering steady state, first order oxygen uptake by cells, spherical geometry and diffusivity independent of the position, these equations summarise in

$$\frac{{\text{d}}^{2}P_{O2}}{{\text{dr}}^{2}}=\frac{k_{R}}{D}P_{O2},$$ (1)

where *k*_*R*_ is the oxygen uptake rate, *D* diffusion coefficient, *P*_o2_ oxygen tension and *r* radius. Let us consider boundary conditions ${\frac{\text{d}P_{O2}}{\text{dr}}|}_{\text{r}=0}=0$ and $P_{O2}{|}_{r=r_{T}}=P_{O2}{}^{max}\left(r_{To},r_{T}\right)$, where P_O2_^max^ is the oxygen tension at peripheral tissues. Here, oxygen at the tumour boundary depends on initial tumour radius $r_{To}=\sqrt[{3}]{\sum V_{o}(t=0)}$ and tumour radius at time *t*, $r_{T}=\sqrt[{3}]{\sum V_{i}(t)},t>0$. Now, we solve by the substitution of variables method, where $z=\frac{{\text{d}}^{}P_{O2}}{{\text{dr}}^{}}$ is an auxiliary variable, thus Eq (1) results in two first order ODEs,

$$\frac{{\text{d}}^{}P_{O2}}{{\text{dr}}^{}}=z,$$ (2)

$$\frac{{\text{d}}^{}z}{{\text{dr}}^{}}=\frac{k_{R}}{D}P_{O2}.$$ (3)

Integrating this with the set boundary conditions we obtain,

$$P_{O_{2}}=C\cdot e^{-\sqrt{k_{R}/D}\cdot r}\left(1+e^{2\sqrt{k_{R}/D}\cdot r}\right),$$ (4)

$$C=\frac{P_{O2}{}^{max}\left(r_{To},r_{T}\right)}{e^{-\sqrt{k_{R}/D}\cdot r_{T}}\left(1+e^{2\sqrt{k_{R}/D}\cdot r_{T}}\right)}.$$ (5)

If we now define the apparent oxygen uptake rate as, $k_{R}\prime =\sqrt{k_{R}/D}$ and remembering the expression for the hyperbolic cosine, cosh(*x*) = (*e* ^*x*^+*e* ^*−x*^)/2, we can simplify it into

$$P_{O_{2}}=P_{O2}{}^{max}\left(r_{To},r_{T}\right)\cdot \frac{\text{cosh}\left(k_{R}\prime \cdot r\right)}{\text{cosh}\left(k_{R}\prime \cdot r_{T}\right)}.$$ (6)

**Fig 2:**
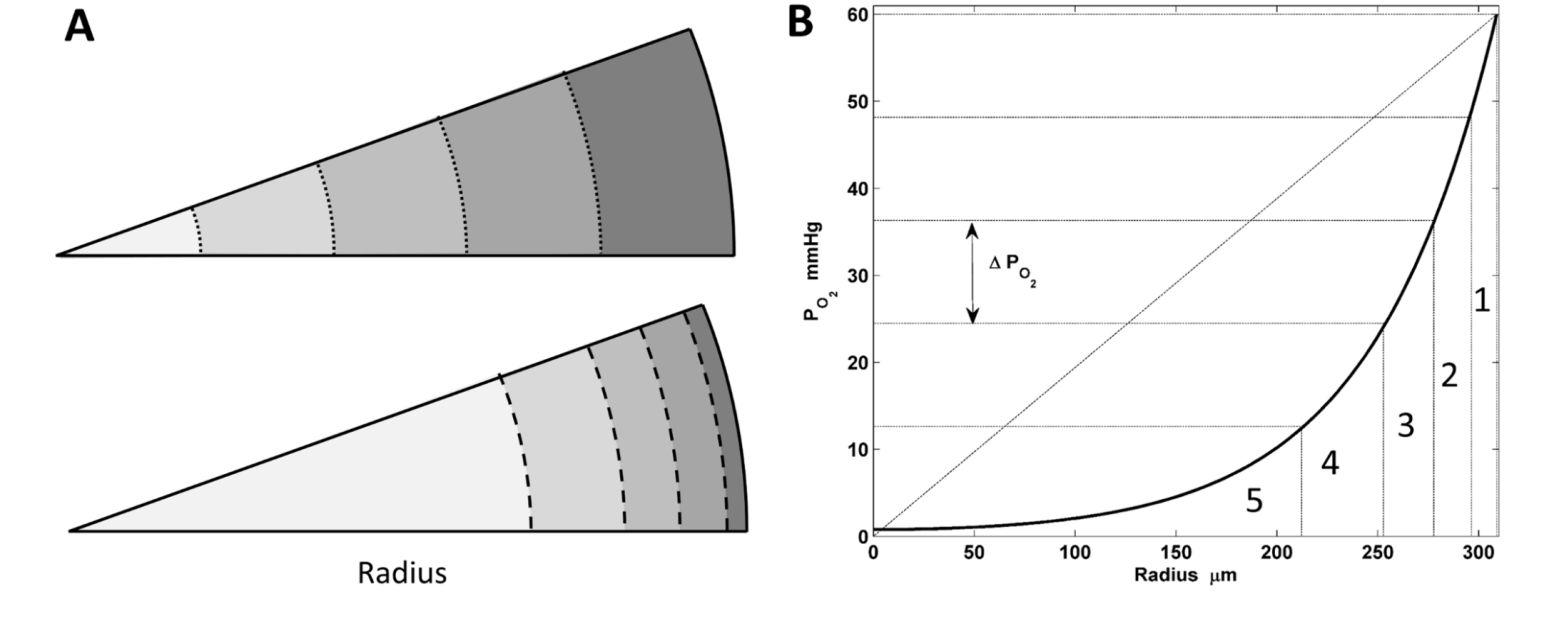
Spatial discretisation. (A) comparison between linear (top) and exponential spatial discretisation (bottom). (B) example of spatial discretisation of 1–5 shells with constant oxygen drop.

The tumour is considered to have a spherical shape, and we discretise space by dividing the tumour into *n* spherical shells, labelled by the index *i* (see Fig 2). The dynamics at each shell are described by its separate set of equations, hence the Eqs (11–14) are labelled by the shell index *i*. Nevertheless, this spatial discretisation is far from trivial. In this case, we chose our spatial discretisation to minimise the error between the spatial distribution of oxygen and assuming constant concentration in each shell. For that reason, we discretised at exponentially -rather than linearly- distributed radii as Fig 2 suggests.

Hereby, the drop of oxygen is constant for the whole tumour at any given time. We achieve that by dividing the total drop of oxygen at a given time ($\Delta P_{O2,Total}={P_{O2}|}_{r_{T}}-{P_{O2}|}_{r=0}$) by the number of shells (*n*),

$$\Delta P_{O2,i}=\frac{\Delta P_{O2,Total}}{n},\;t,\forall i.$$ (7)

Now, solving for Eqs (7) and (6) we obtain the following expression for the oxygen drop

$$\Delta P_{O2,i}=\frac{P_{O2}{}^{max}\left(r_{To},r_{T}\right)}{n}\left(1-\frac{1}{\text{cosh}\left(k_{R}\prime \cdot r_{T}\right)}\right).$$ (8)

Since the oxygen relationship is now linear with *i*, the oxygen at each consecutive shell can be defined from inside-to-outside or outside-to-inside. We arbitrarily chose to add up shells from the innermost shell, resulting in

$$P_{O2,i}=P_{O2}\left(r=0\right)+\text{i}\cdot \Delta P_{O2,i}=P_{O2}{}^{max}\left(r_{To},r_{T}\right)\cdot \left(\frac{1}{\text{cosh}\left(k_{R}\prime \cdot r_{T}\right)}+\frac{i}{n}\left(1-\frac{1}{\text{cosh}\left(k_{R}\prime \cdot r_{T}\right)}\right)\right).$$ (9)

Now, the concentration of the oxygen at the tumour boundary was considered to reduce with the exposed surface area of the spherical tumour,

PO2max(rTo,rT)=PO2max⋅(VToVT)2/3, (10)

with $V_{To}=\sum^{\text{​}}V_{i}(t=0)$, $V_{T}=\sum^{\text{​}}V_{i}(t)$ and oxygen at tumour periphery *P*_o2_^*max*^. Eqs (9) and (10) give the final equation for oxygen diffusion (Eq (12)).

Hypoxia and necrosis arise as a direct consequence of insufficient oxygen distribution [41]. We consequently describe them as “smooth switches” [42], described by saturable (sigmoid, or “S” shaped) functions (similar to Michaelis-Menten equations, Eqs (13) and (14)). This means that at a particular oxygen concentration, cells will become stressed (hypoxia), which at a further stage will become necrotic. This is not set as a threshold (step (Heaviside) function) but as a smooth probabilistic process, so that at any point in the tumour there is a non-zero probability to find any type of cell: normoxic, hypoxic or necrotic. For more information on the derivation of the equations please see S1 Text. Also, a Matlab code script can be found in S2 Text.

### Summary of main equations

The main ODM equations derived from Eqs (1–10) are summarised below,

$$\frac{dV_{i}}{dt}=k_{p}\cdot P_{O_{2},i}\cdot V_{i},$$ (11)

PO2,i=PO2maxn⋅cosh(kR′⋅rT)(VToVT)2/3(n−i+i⋅cosh(kR′⋅rT)), (12)

$$V_{i}^{H}=\frac{P_{O_{2},i}}{K_{H}+P_{O_{2},i}}\cdot V_{i},$$ (13)

$$V_{i}^{N}=\frac{P_{O_{2},i}}{K_{N}+P_{O_{2},i}}\cdot V_{i}.$$ (14)

These equations are sufficient to describe the ODM, which is represented graphically in Fig 1A. The volume of each tumour shells (*V*_*i*_) is calculated via Eq (11), which is the equation governing tumour growth, while Eq (12) is used to calculate the oxygen profile across the tumour shells. The volume of the total tumour is the arithmetic sum of the shells, (*V*_*T*_ = Σ_*i*_ *V*_*i*_). Last, Eqs (13) and (14) are twin expressions to calculate volumes of hypoxic and necrotic regions respectively. A quick reference guide to the mathematical notation can be found in Table 1.

Tumour division is controlled by *k*_*p*_ and the oxygen levels, which are calculated in the radial direction for each time step. As a consequence, regions of low oxygen are hypoxic; regions of no oxygen are necrotic.

### The ODM fits growth curves, hypoxia and necrosis data identifiably

**Fig 3:**
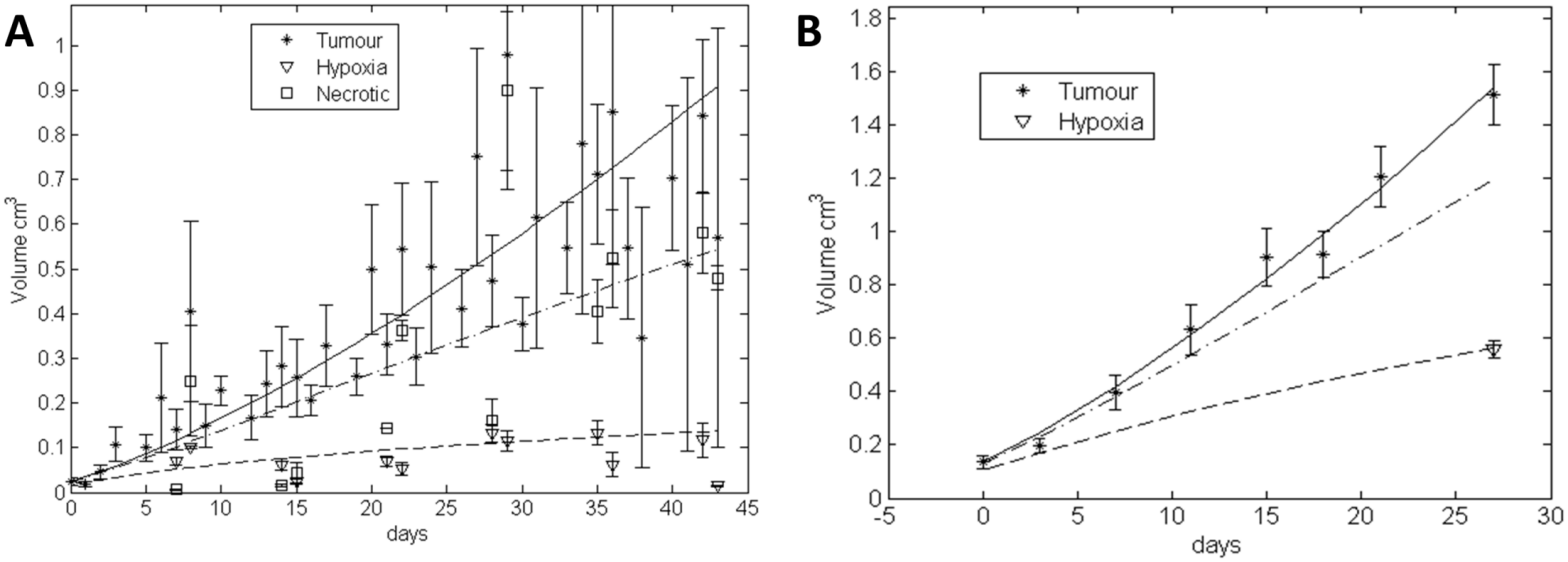
Complete model fit ODM. (A) Results of the fit with data (extracted from paper by Ribba’s group [5]). The plot contains information for tumour volume, hypoxia and necrosis for colon carcinoma cell line HT29. (B) Model fit for a data set containing IHC information on HIF1α for hypoxia at end of study point for MCF7 tumours.

To validate the ODM, we used data published by Benjamin Ribba et al. [5] (see Fig 3A). In this manuscript Ribba and colleagues developed an Odinary Differential Equation (ODE) logistic model addressing necrosis, hypoxia, vessels and proliferating fractions. For the validation, they utilised a single study of 15 athymic mice implanted subcutaneously with HT29 cells (Fig 3A). Tumours were frequently measured and two to three mice were euthanized weekly for immunohistochemical (IHC) analysis (necrosis and hypoxia) [5].

Our model prediction results are similar to those obtained by Ribba et al. The parameter values denoted reasonable standard errors (see Table 2). We assessed the identifiability through the collinearity index ($\gamma =\frac{1}{\sigma_{Last}}$) and condition number ($\kappa =\frac{\sigma_{1}}{\sigma_{Last}}$), where *σ*_*last*_,*σ*_1_ are the largest and smallest values of the diagonal of the factorised normalised sensitivity matrix (see S1 Text and [43]). Briefly, the sensitivity matrix (S) will be normalised (S~=S∙θ~Y~) and then factorised ($\tilde{S}=U∙\Sigma ∙V^{T}$), which diagonal of the factorised matrix (*diag*(Σ)) contains the elements *σ*_*Last*_,*σ*_1_. The values of collinearity index and condition number show that the system is locally identifiable (see S1 Text), are below the set thresholds (see Table 2). The rank of the Fisher Information Matrix (FIM) is 4, which means that all 4 parameters are practically identifiable (Table 2). Finally, the cost function or objective function (OF) was defined as a least-squares weighed sum between necrotic, hypoxic and proliferating regions,

minVOF=∑t∑r(V˜t,r−Vt,r)Nr (15)

Where ${\tilde{V}}_{t,r}$ and *V*_*t*,*r*_ represent the estimated and measured volumes at each time point t and at each tumour region *r* = {*T*,*H*,*N*}. *N*_*r*_ is the number of data points available for each region. In the tables the residual is expressed as the solution of Eq (15).

This data fit demonstrated that the ODM describes, with sufficient accuracy, the hypoxic and necrotic regions. However, in practice these data sets are difficult to obtain, because it requires the sacrifice of animals at each time point in order to harvest the tumour for histological analysis.

**Table 2:** Parameter results (*k*_*P*_, $k_{R}^{\prime }$, *K*_*H*_, *K*_*N*_) for the ODM model for Ribba et al. [5]. Data and MCF7 with hypoxic endpoint.

		Ribba 2010	MCF7
*k*_*p*_	**(mmHg x day)** ^**-1**^	0.0058	0.0027
*SE*(*k*_*p*_)	**(mmHg x day)** ^**-1**^	0.0041	0.0027
$k_{R}^{\prime }$	**cm** ^**-1**^	13.37	7.54
$SE\left(k_{R}^{\prime }\right)$	**cm** ^**-1**^	2.39	1.90
*K*_*H*_	**mmHg**	25.9	13.8
*SE*(*k*_*H*_)	**mmHg**	54.0	27.3
*K*_*N*_	**mmHg**	20.2	-
*SE*(*k*_*N*_)	**mmHg**	3.8	-
Rank	**-**	312	342
Y	**-**	49	35
k	**-**	4	3
Residual	**-**	0.18	0.09

Online imaging studies of hypoxia using PET tracers can efficiently record multiple time points [44] of hypoxia, however, they depend upon immobilisation of the animal and are expensive. To meet economic requisites, some studies are designed to obtain one single hypoxia measurement per tumour at the end of study. One time point is not sufficient to fit a model with confidence. Nevertheless, it can provide an estimate of the parameter range. As an in house example, we used a study in the breast cell line MCF7 with a single end-of-study hypoxic point (from HIF1α staining). The ODM fits the data identifiably even though the data set has been substantially reduced (see Table 2 and Fig 3B).

### Faster vs Slower Growing Xenografts differ in growth rate, necrosis, vessels and stroma

**Fig 4:**
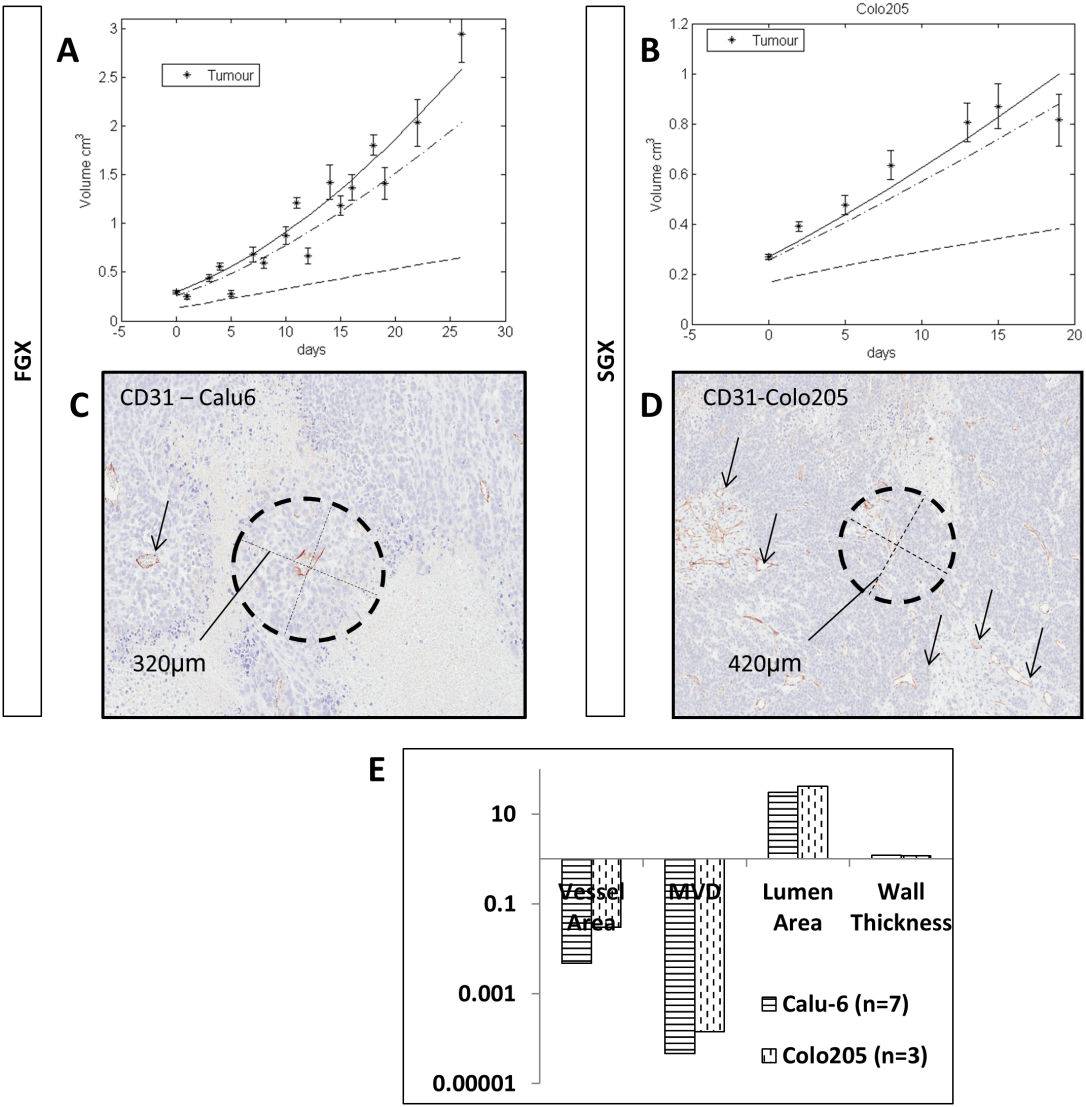
Growth curve fit for 2 example xenografts. Panels (A-B) show fits of the model for Calu6 and Colo205. The plots also include simulation of hypoxia and necrosis. (A) is a faster proliferating tumour model (Calu6) and (B) grows slightly slower (Colo205). (C-D) CD31 IHC staining in Calu6 and Colo205 respectively. (E) summary data of CD31 for both tumour models.

Motivated by the observed doubling times in different models (xenograft ~1week, explant ~weeks and human ~months or years), we divided the cell lines into **F**aster and **S**lower **G**rowing **X**enografts (FGX and SGX respectively, see Fig 4A–4D). The growth rate was windowed by dividing the ranges of tumour growth across all cell lines and dividing it into SGX<0.05cm^3^/day and FGX>0.05cm^3^/day. Fits to xenograft data were better than in explants (Fig 4A and 4B). Note that the last data point presented in Fig 4B shows a logistic trend. This behaviour is captured in the ODM by means of the oblique asymptote dictated by the parameters *k*_*p*_ and $k_{R}\prime$, however there are restrictions to the complete saturation (see model in S1 Text for a more diverse model).

We investigated the relationship between pathophysiology and growth rate in Colo205 as a SGX (Fig 4D) and Calu6 as a FGX (Fig 4C). In general, more stroma is recruited in SGX and necrosis appears further away from the vessels (Fig 4C and 4D). Mean vessel density (MVD), vessel and lumen area in Calu6 showed reduced values compared to Colo205 (Fig 4E), suggesting there is a relationship between pathophysiology and growth rate in xenografts, also observed across explants and the clinic (Fig 6C).

### The growth patterns of forty two cell lines and five explant-like models are described by different ODM parameter values

After validation of the ODM dynamics with rich datasets, we explored a larger range of cell lines with restricted data. We fitted the ODM to control data from 38 cell lines from various pre-existing projects (see Table 3). However, in this case, hypoxia and necrosis data were not available, therefore we fixed the parameters *θ*_*f*_ = {*K*_*H*_,*K*_*N*_} ∈ ℜ^2^ to the values observed in Fig 3 (the system would be insensitive to the parameters and thus non identifiable). Our selected subset of parameters is then $\hat{\theta }=\left\{k_{p},k_{R}^{\prime }\right\}\in {ℜ}^{2}$, which will provide an idea of the variable interplay between delivery and cell line intrinsic factors over a series of models.

**Table 3:** Parameter results (*k*_*p*_ and $k_{R}^{\prime }$) for the ODM model in xenografts. Confidence intervals for each parameter are specified as well as the rank of the FIM (as a measure of the number of identifiable parameters), the collinearity index (Identifiable if γ<10), the condition number (Indentifiable if κ<1000) and the normalised residual. Cell lines denoted by * are unidentifiable and cell lines denoted by ^#^ are arguably identifiable.

			*k*_*p*_	*SE*(*k*_*p*_)	$k_{R}^{\prime }$	$SE\left(k_{R}^{\prime }\right)$	ΔV	κ	γ	Rank	Residual
			(mmHg x day)^-1^	(mmHg x day)^-1^	cm^-1^	cm^-1^	cm^3^/day	-	-	-	-
**Repr. Sys.**	**Lung**	Calu6*	0.0001	0.0018	5.06	<0.01	0.10	529500	17874	2	0.20
	**Colon**	**Colo205**	0.0005	0.0014	3.17	2.24	0.03	46	9	2	0.10
	**Lung**	**H460**	0.0041	0.0035	13.38	3.08	0.14	135	10	2	0.30
		**H1975** ^**#**^	0.0013	0.0020	6.05	2.21	0.05	43	21	2	0.06
		**H3255**	0.0001	0.0012	2.83	17.08	0.03	6	3	2	0.02
		**A2058**	0.0005	0.0030	6.26	16.02	0.07	8	4	2	0.07
		**PC9**	0.0003	0.0008	8.23	41.11	0.03	3	1	2	0.15
		**A549b**	0.0001	0.0026	3.22	22.90	0.06	11	2	2	0.03
	**Breast**	**HCC1954** ^**#**^	0.00005	0.0004	1.40	2.40	0.06	19	15	2	0.01
	**Cervix**	**ME180** ^**#**^	0.0003	0.0014	3.20	3.39	0.03	14	21	2	0.02
**Gastrointestinal**	**Gastric**	**SNU5** ^**#**^	0.0004	0.0011	3.35	2.32	0.04	56	17	2	0.04
		**SGC31**	0.0002	0.0015	6.59	34.59	0.05	8	4	2	0.03
		**SGC37**	0.0005	0.0031	8.51	46.99	0.01	9	1	2	0.15
		**SGC70**	0.0008	0.0045	11.64	28.99	0.05	13	2	2	0.21
		**SGC71**	0.0005	0.0043	7.46	29.73	0.08	11	2	2	0.08
		**SGC100**	0.0002	0.0041	3.06	53.31	0.08	7	1	2	0.02
		**SGC161** ^**#**^	0.0005	0.0016	3.33	2.63	0.04	31	28	2	0.02
		**SGC181** ^**#**^	0.0005	0.0019	2.69	2.58	0.03	35	32	2	0.01
		**SGC184** ^**#**^	0.0002	0.0028	4.22	5.70	0.03	89	19	2	0.03
		**MKN45** ^**#**^	0.0006	0.0013	4.03	2.49	0.05	64	18	2	0.03
		**HS746T**	0.0007	0.0050	9.68	36.93	0.04	9	1	2	0.15
	**Colon**	**MC38** ^**#**^	0.0043	0.0047	17.19	3.94	0.13	93	15	2	0.11
		**Lovo**	0.0009	0.0040	14.87	26.75	0.24	10	2	2	0.23
		**HCT116**	0.0002	0.0029	4.93	40.20	0.11	10	1	2	0.07
		**HT29** ^**#**^	0.0011	0.0021	4.30	2.23	0.06	84	16	2	0.04
**Other**	**Skin**	**A375** ^**#**^	0.0022	0.0018	11.71	2.88	0.06	139	12	2	0.19
	**Bladder**	**MGHU3** ^**#**^	0.0035	0.0036	7.81	1.99	0.09	77	12	2	0.26
	**Kidney**	**RCC47**	0.0002	0.0003	6.85	3.47	0.00	4	11	2	0.51
		**786O** ^**#**^	0.0004	0.0007	4.68	2.31	-0.01	33	14	2	0.06
	**Blood**	**KMS11**	0.0002	0.0022	5.85	35.80	0.07	11	1	2	0.08
	**Lymph**	**HT1080**	0.0030	0.0028	8.52	1.98	0.07	78	10	2	0.36
		**Ri1**	0.0002	0.0027	3.32	34.28	0.07	6	1	2	0.06
		**RS411**	0.0003	0.0024	12.42	25.71	0.12	16	2	2	0.18
		OCLy10*	>100	-	>100	-	0.03	>10000	>1000	2	0.04
		**OCLy19**	0.0002	0.0044	4.17	25.02	0.12	17	2	2	0.04
		**A20**	0.0003	0.0004	7.31	2.55	0.01	14	5	2	0.57
	**Mouse**	**CT26**	0.0003	0.0032	3.07	33.56	0.03	9	2	2	0.09
	**Stem cell**	**ECB1**	0.0001	0.0011	1.63	27.49	0.04	4	1	2	0.04

**Fig 5:**
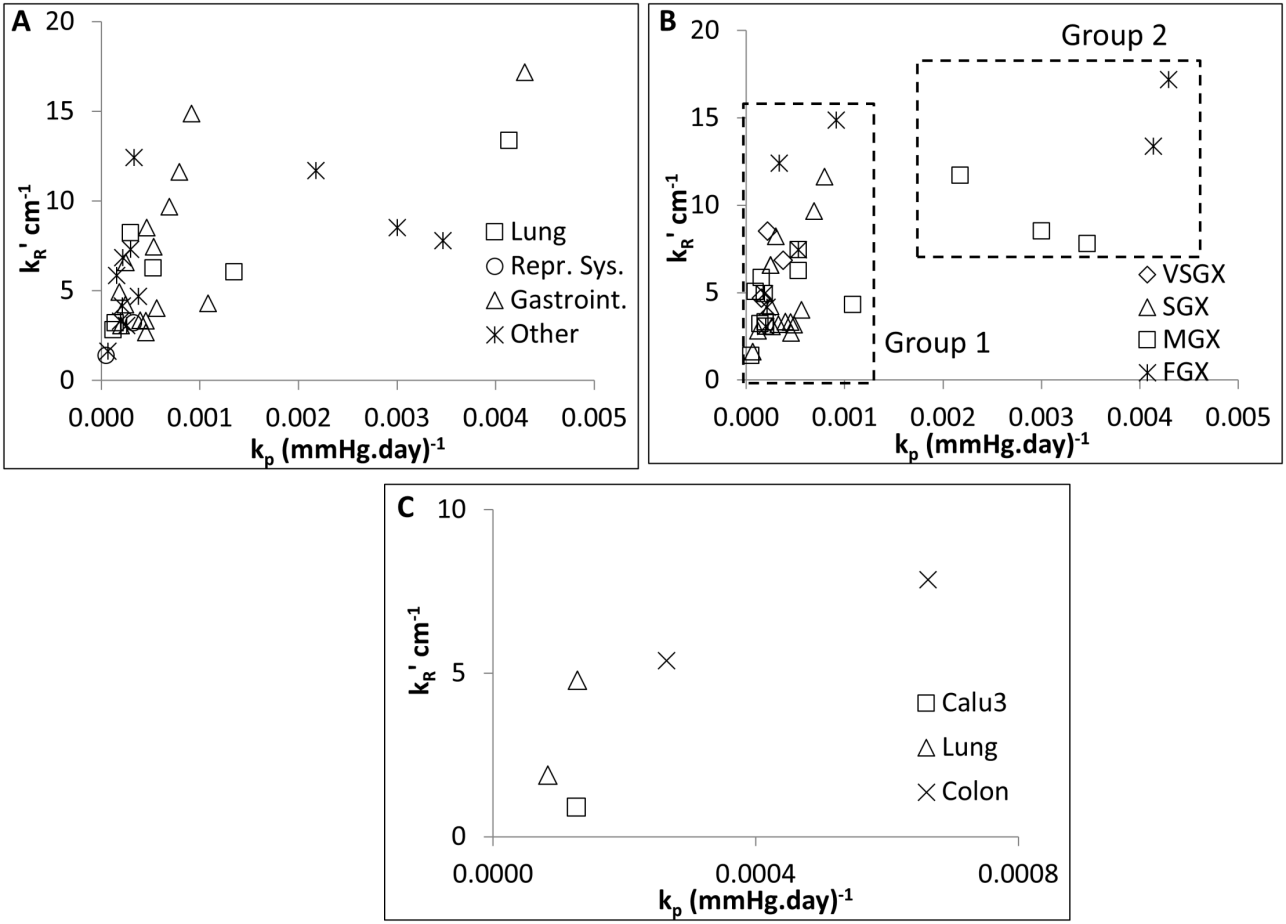
Results of parameter estimates. (A) Parameter space (*k*_*p*_-$k_{R}^{\prime }$) for different tissue of origin lines. (B) Same as (A) but divided into Fast, Medium, Slow and Very Slow Growth Xenografts. (C) Parameter space for explant-like tumour models.

Parameter values for the proliferating rate lie in the range *k*_*p*_ = 0.0001–0.0043*mmHg* ^−1^∙*day* ^−1^ and in the range $k_{R}^{\prime }=2-17cm^{-1}$ for the oxygen uptake rate (see Fig 5A). Standard errors are always comparable to the parameter values, suggesting that there is inter-animal variability. In those cases a mixed effects model would be advisable.

We note the collinearity index and condition number are well within the identifiable range. Residual errors show appropriate goodness of fit. We identified a posteriori 2 groups of parameters within the dashed boxes. ***Group 1*** parameters cluster in the lower range of *k*_*p*_ and demonstrate a wider spread over $k_{R}^{\prime }$, this group contains the slowest growing cell lines. Besides, $k_{R}^{\prime }$ parameter values in ***Group 2*** are found dispersed around an average of 12 cm^-1^, whereas *k*_*p*_ demonstrates large range of values; we identify this group with the faster growing cell lines (Fig 5B). However, the tissue of origin showed not to be predictive of the tumour growth rate.

Regrouping the cell lines in terms of growth rates, we found that *k*_*p*_ is often the main driver of growth speed, whereas $k_{R}^{\prime }$ seem to vary across a range of values. The medians for both parameters are 0.0023 mmHg^-1^ day^-1^ and 16.0 cm^-1^ respectively.

The explants used here and the cell line Calu3 present with a stromal vessel (SV) phenotype (as described by Smith and co-workers [15]), thus we referred to them as **explant-like models in the present manuscript**. This phenotype is more complex than typical xenografts (Tumour Vessel phenotype [15]) and more reliably resembles the stromal complexity the clinical carcinomas of interest, where a mature vasculature surrounded by pericytes and myofibroblasts is commonly found. The explant-like tumours range *k*_*p*_ = 1–7∙10^−4^∙day^−1^∙mmHg^−1^ (in the range of very slow growing xenografts) and $k_{R}^{\prime }=0.9-7.8cm^{-1}$(similar to the xenografts, see Fig 5D) with the exception of colon 1.

As an exception, some cell lines (Calu6 and OCLy10) were not practically identifiable (see Table 3); other cell lines were poorly identifiable (786O, A375, MGHU3, HT29, MC38, MKN45, SGC161, SGC181, SGC184, ME180, H1954, H1975). This was partly because of the quality and quantity of the data (some of these datasets include only 2 data points or contain experimental errors). Parameter values are within ranges observed for the other cell lines, but the confidence intervals are very wide.

### ODM does not always succeed in describing the growth pattern of tumours with more complex pathophysiologies

**Fig 6:**
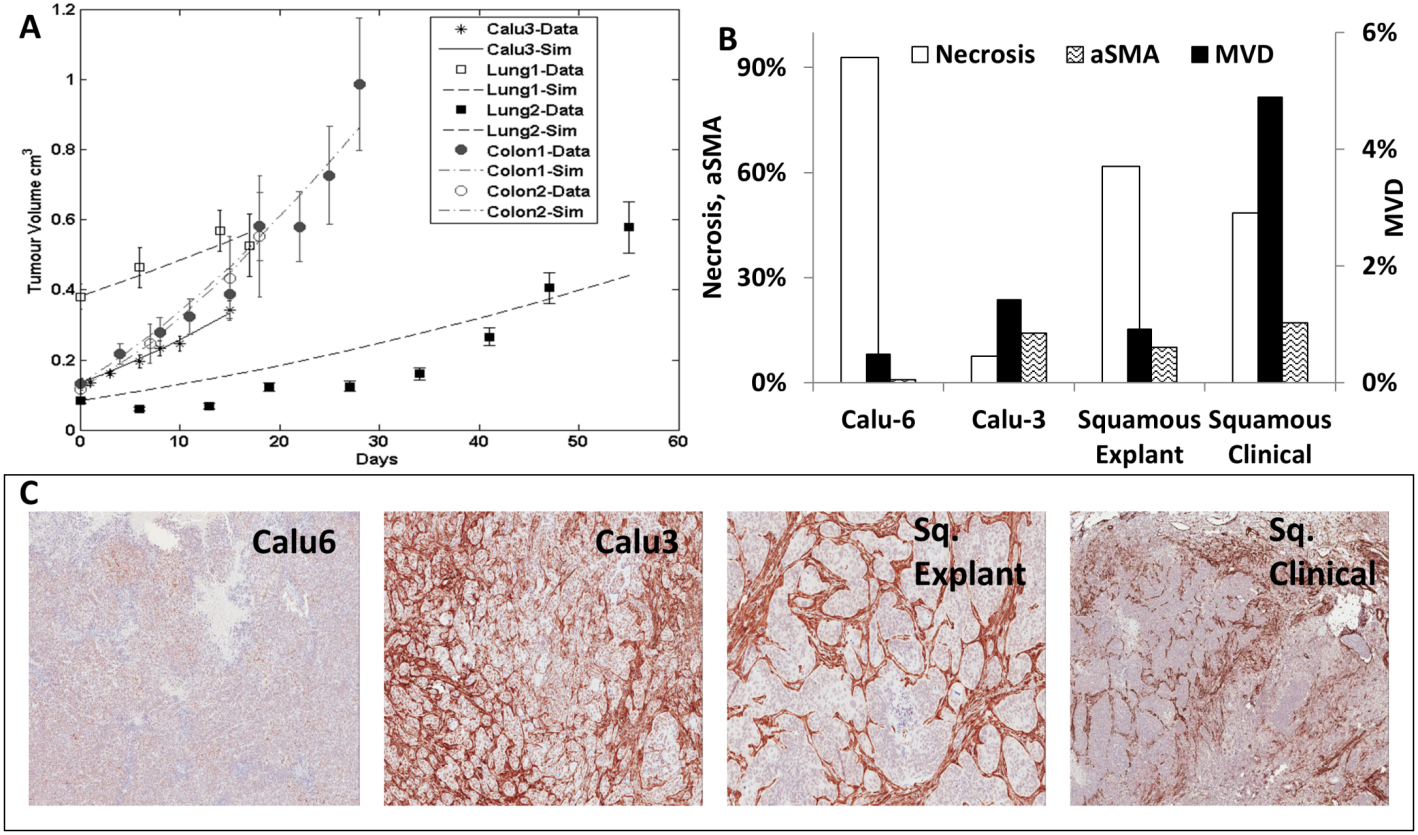
Growth curve fit for explants. (A) Growth curve fit for 4 explant models, 2 for squamous lung carcinoma and 2 for colorectal carcinoma. The xenografted cell line Calu3 shows very similar behaviour to explant models. (B) Comparison between Calu6 and Calu3 lung cell lines; a squamous lung cancer explant; and clinical tumour material analyses. The bar chart shows the proportions of microvessel density (MVD) in area (quantified from CD31), necrosis (quantified from Hematoxylin & Eosin staining) and stroma (alpha smooth muscle actin (αSMA) positive staining). (C) Images of the different tumour models stained for αSMA and counter-stained with hematoxylin.

In an effort to extend the application of the model, we applied the ODM to five explant-like models. These models can be very heterogeneous in pathophysiology, an effect that may also be evident in the growth curves. Results of the model-data fit are acceptable, but generally worse than in xenografts (Fig 6A). However, *lung 2 explant* is poorly fitted on a point by point basis.

At this stage, growth curves alone are insufficient to understand pathophysiological differences between preclinical and clinical models. We stained tissue sections of the following tumours: Calu6 (n = 8), Calu3 (n = 7), one squamous cell lung cancer explant (n = 28) and squamous cell lung clinical cancers (n = 12) from AstraZeneca databanks. Both cell lines are derived from non-small cell lung cancer. We observed that the microvessel density (MVD) increases steadily from 0.4% in Calu6 to 4% in clinical tumours. We also looked at the tumour-associated stromal cells (stained with αSMA), i.e. pericytes and myofibroblasts. These are the main stromal cells contributing positively to tumour development. We highlight the 0.8% of αSMA staining in Calu6 versus a range between 10–17% in the other models (Fig 6B), over 1 order of magnitude lower (also observed in Fig 6C). Lastly, the appearance of necrosis decreases from Calu6 to clinical tumours, with the exception of Calu3. This analysis of histological data (Fig 6) allows us to identify that even if the growth curves are similar, the tumour composition might play an important role in the tumour behaviour and treatment. Further, the very large standard error observed in Table 4 for $k_{R}^{\prime }$, demonstrate that the complex growth dynamics introduced by the tumour microenvironment are captured by the oxygen uptake rate ($k_{R}^{\prime }$).

**Table 4:** Parameter results (*k*_*p*_ and $k_{R}^{\prime }$) for the ODM model in explant-like tumours.

	*k*_*p*_	*SE*(*k*_*p*_)	$k_{R}^{\prime }$	$SE\left(k_{R}^{\prime }\right)$	ΔV	κ	γ	Rank	Residual
	(mmHg x day)^-1^	(mmHg x day)^-1^	cm^-1^	cm^-1^	cm^3^/day	-	-	-	-
**Calu3**	0.0001	0.0014	0.90	64.90	0.0007	10	11	2	<0.01
**Lung 1**	0.0003	0.0021	5.38	71.27	0.0005	6	1	2	0.10
**Lung 2**	0.0001	0.0003	4.78	2.45	0.0008	12	42	2	0.01
**Colon 1**	0.0007	0.0023	7.86	38.37	8.711	7	8	2	0.04
**Colon 2**	0.0001	0.0009	1.88	54.24	0.0096	9	3	2	0.03

### Introduction of stroma in the ODM may be used to describe tumour-stroma interaction

**Fig 7:**
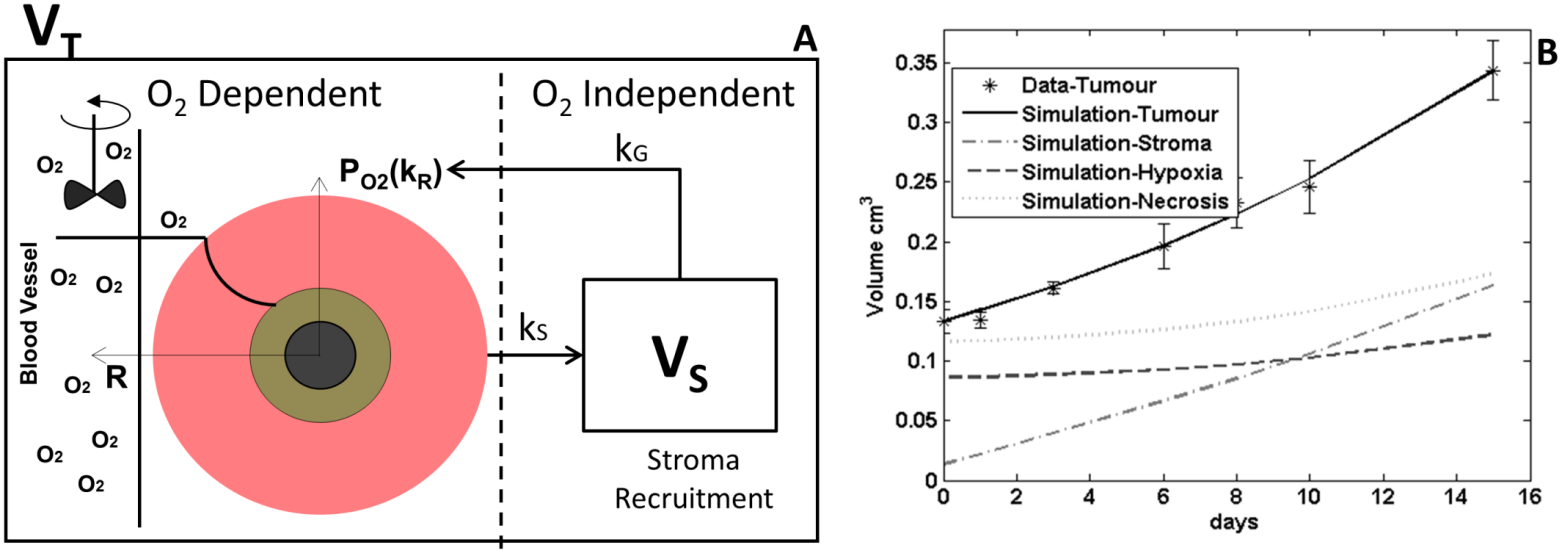
ODM adaptation. (A) Sketch of the ODM model (oxygen dependent, left) as described here plus a single compartment (Stroma) (V_S_, oxygen independent, right). Where k_S_ is the stroma recruitment constant and k_G_ is the growth enhancement constant. (B) Example of cell line fit for Lung 1 explant model. Tumour volume over time and hypoxia at end of study are presented. Also a simulation of necrosis is presented.

As showed in the histological data (Fig 6), clinical tumours have a complex microenvironment, very different to the xenografts. One of the main differences observed is the tumour stroma. Therefore, we adapted the model by adding one compartment for the formation of stroma (Fig 7A).

Initially, the stroma is recruited from distant sites of the body and is partly triggered by the immune response. The connection between the desmoplastic (stroma formation) and angiogenic (blood vessel formation) reactions has been identified in a series of animal models and clinical metastases [45], but not accurately understood. As a first approximation, we assumed that stroma by default carries vessels. Also, cancers trigger an inflammatory response, quickly recruiting cells from distant sites of the body [40], similar to a “wound that never heals”. Hence, we considered that the stroma is recruited proportionally to the volume of the “wound/cancer” (*V*_*T*_) with proportionality constant k_s_. This assumption requires thorough biological support and refinement.

The stroma also triggers a modification in the proliferation rate *k*_*p*_(*mmHg* ^−1^∙*day* ^−1^), which turns into *k*_*PG*_(*mmHg* ^−1^ ∙*day* ^−1^ ∙*cm* ^−3^) = *k*_*P*_∙*k*_*G*_. This assumption stems from one of the hallmarks of cancer, stating that tumour-associated stroma provides the key paracrine signals for tumour development [46]. With these modifications, the model Eq (11) becomes Eq (16), while Eq (17) is added.

$$\frac{dV_{i}}{dt}=k_{PG}\cdot P_{O_{2},i}\cdot V_{i}\cdot V_{S}$$ (16)

$$\frac{dV_{S}}{dt}=k_{S}\cdot \sum V_{i}$$ (17)

We fitted this model to the Calu3 data explant, showing very good fit (parameters for hypoxia and necrosis remain fixed). Stroma constituted around 30% of the tumour volume in the prediction. However, this is a marginally identifiable case with very wide confidence intervals (Table 5).

**Table 5:** Parameter results (*k*_*PG*_, $k_{R}^{\prime }$, *k*_*S*_) for the ODM model for Lung 1 explant model data.

*k*_*PG*_	SE(*k*_*PG*_)	$k_{R}^{\prime }$	$\text{SE}\left(k_{R}^{\prime }\right)$	*k*_*s*_	SE(*k*_*S*_)	Rank	γ	κ	Residual
(mmHg x day x cm^3^)^-1^		cm^-1^		mmHg		-	-	-	-
0.005	0.154	23.7	84.9	0.07	0.05	3	85	16122	0.04

## Discussion

We have demonstrated that large data sets for hypoxia and necrosis along with tumour volume are uniquely fitted with 4 parameters. This is a small number compared to other models, e.g. 6 parameters in the model by Ribba et al. [5]. In practice, data sets may not be as rich, having reduced number of time points describing hypoxia. However, we typically find end-of-study analysis of hypoxia and necrosis, which can be uniquely identified (Fig 3B), but arguably used. To date, most models found in preclinical oncology are either empirical or implement non-allocated compartments to describe hypoxic/necrotic regions. In the ODM, the calculated continuous spatial oxygen distribution dictates growth, hypoxia and necrosis, key physiological parameters. This may be easily extrapolated to drug studies.

Growth rates increase in a quasi-monotonic fashion from the clinic, through to explants, into xenografts. Typically, slower proliferating tumours develop more stroma and the vasculature is more mature (Fig 6C). These tumours are, in general, also less necrotic. This is, although a generalisation based on a few observations. Though, we hypothesise this could apply to many cell lines, this has not been fully explored yet and we encourage other scientists to investigate it.

We have aimed for a more informative model using pathophysiological assumptions, however, the interpretation of the parameters can be further discussed. We postulate that the model parameters signify:

*K*_*H*_, *K*_N_—hypoxia and Necrosis switch constant. These constants account for the variability in the transition to hypoxia and necrosis;
*k*_*p*_—proliferation rate constant: natural frequency of cell division, in other words an *in vivo* expression of the *in vitro* doubling time (free of delivery burdens). We expect *k*_*p*_ to capture genetic properties of the cell to commit to the cell cycle;
$k_{R}^{\prime }$—oxygen uptake rate: oxygen needed by each cell to divide. In our definition $k_{R}^{\prime }$ should capture the microenvironment characteristics. It may also account for angiogenesis, in other words the “apparent oxygen reach”. In short, $k_{R}^{\prime }$ angiogenic values (1–70 cm^-1^) are much lower than the avascular hypothetical calculations (~200–400 cm^-1^ (Table 2)). This means that oxygen appears to reach further than reported in the literature. This may be identified with angiogenesis (see Fig F in S1 Text).

Since both parameters describe different aspects of the tumour physiology, we have aimed to group the parameter space according to the sensitivity of both parameters, finding only very weak associations and no differential affinity to organ of origin (e.g. lung) or cell type (e.g. carcinoma).

On the one hand, ***Group 1*** with a range of values of $k_{R}^{\prime }$ and low *k*_*p*_, suggests that microenvironment plays a major role in the development of these tumours. Also, their growth rates are slow. Similarly, explants demonstrate almost identical parameter values, being indicative of growth, but not of physiology.

On the other hand, ***Group 2*** with a $k_{R}^{\prime }$ mean very close to the median values, indicates that most medium to fast proliferating cell lines are better perfused and the tumour matrix is mainly a mixture of sparse stromal and vascular cells. From our experience, these fast growing cells trigger angiogenesis faster, but the immaturity of the vessels, combined with the competition for space of the fast growing cells, leads to occluded vessels, which trigger unexpected necrosis (IHC example shown on Fig E in S1 Text). These observations need to be revisited and supported with further evidence.

Explant-like models, which more closely model clinical pathophysiology, provided poor fits to the ODM (Fig 6A). We suggest incorporating stroma and vasculature in the model to address this problem. Evidence indicates that slower growing models, such as explant-like (also referred as SV) tumours, rely on the recruitment of stroma to grow, followed by reduced necrosis and significantly greater MVD (as elucidated by the results, Fig 6B and 6C). The ODM assumes a simple spherical morphology, which can mimic semi-vascular tumour nests in explant-like tumours. In future versions, we will capture stromal morphology from IHC images and describe it spatially in a 3D version of the ODM. Despite the actual limitations of the ODM so far, xenografts are properly described. The ODM, being a very simple model, captures several aspects of the tumour pathophysiology in xenografts, namely proliferating rate, blood effective perfusion, hypoxia and necrosis. However, the ODM fails to describe explant models fully. This will be amended in more advanced versions of the ODM, which will mimic complex tumour-stromal interactions. This will increase our understanding of animal models, thereby enhancing preclinical decision making and pharmacokinetic and pharmacodynamic (PKPD) predictions in humans.

## Materials and Methods

### Ethics statement

All studies were conducted in accordance with UK Home Office legislation, the Animal Scientific Procedures Act 1986 (ASPA) and with AstraZeneca Global Bioethics policy. The analysis in this paper is retrospective, utilising control/untreated animals of different oncology projects within AstraZeneca between 2004 and 2013. All experimental studies have gone through the AstraZeneca Ethical Review Process. All tumour volumes, animal weights and welfare were maintained within the margins fixed by UK and European regulations. No data was generated specifically for this manuscript.

### Animals and cell lines

We used data from 38 xenografted cell lines implanted in the SCID and nude mice of both sexes. Briefly, 1 x 10^6^ to 1 x 10^7^ human cancer cells, with or without Matrigel, were implanted subcutaneously on the mouse flank. Tumour volumes were calculated from bisecting calliper measurements using the prolate spheroid approximation formula [47]. Tumours were measured 1–3 times weekly.

### Parameter estimation

The optimisation was done with a least-square multiple start global optimiser. The 150 initial estimates for the simulation were selected randomly within a feasible parameter space by the latin hypercube rule.

### Differential-Algebraic Equation (DAE) solver

We solved our deterministic model Eqs (11–14) with a marquart-leuven integrator for stiff ode solvers (matlab, ode15s). The Jacobian was calculated by direct analytical derivation of the DAEs.

### Sensitivity methods

We analysed all possible aspects of the model comparing and contrasting its structural and practical identifiability, testing a variety of scenarios. We applied a Taylor Series approach to evaluate structural identifiability [27,48] to ensure that there was a unique correspondence between model parameter values and the model prediction over time. For practical identifiability, we applied a model-based method calculating collinearity index and condition number [43], broadly validated for various mathematical biology applications [49]. The standard errors were calculated with the covariates (diagonal of the covariance matrix, where *Co* = *FIM* ^−1^), being Co the covariance matrix and FIM the Fisher Information Matrix (*FIM* = *S* ^−1^ × *S*).

### Histopathology and image processing

The morphologies of explants and clinical tumours were determined from formalin-fixed paraffin-embedded tissue sections. The tumour sections were then stained for CD31, αSMA, CC3 or HIF1α, counter-stained with Carazzi’s hematoxylin, and subsequently scanned. Images have been colour deconvoluted to highlight the main features (stroma and epithelium). Finally, the images were quantified with Aperio Microarrays, Genie and ColorDeconvolution algorithms.

### Software and hardware

We used a Windows 7, Intel Exon (R) CPU E5-26200@ 2 x 2 GHz with Matlab 2013b (Mathworks, Massachusetts, USA) and the global optimisation toolbox.

## Supporting Information

S1 Text Remarks on the mathematical formulation of the model and extra comments to the results. (PDF)

S2 Text Script with the ODE model. (M)

## References

[1] Bernard A, Kimko H, Mital D, Poggesi I. Mathematical modeling of tumor growth and tumor growth inhibition in oncology drug development. Expert opinion on drug metabolism & toxicology. 2012;8(9):1057–69. 22632710 10.1517/17425255.2012.693480

[2] Roose T, Chapman SJ, Maini PK. Mathematical models of avascular tumor growth. Siam Review. 2007;49(2):179–208.

[3] Araujo R, McElwain D. A history of the study of solid tumour growth: the contribution of mathematical modelling. Bulletin of mathematical biology. 2004;66(5):1039–91. 15294418 10.1016/j.bulm.2003.11.002

[4] Stroh M, Duda D, Takimoto C, Yamazaki S, Vicini P. Translation of Anticancer Efficacy From Nonclinical Models to the Clinic. CPT: pharmacometrics & systems pharmacology. 2014;3(8):e128. 25098530 10.1038/psp.2014.28 PMC4150926

[5] Ribba B, Watkin E, Tod M, Girard P, Grenier E, You B, et al A model of vascular tumour growth in mice combining longitudinal tumour size data with histological biomarkers. European Journal of Cancer. 2011;47(3):479–90. 10.1016/j.ejca.2010.10.003 21074409

[6] Frieboes HB, Wu M, Lowengrub J, Decuzzi P, Cristini V. A computational model for predicting nanoparticle accumulation in tumor vasculature. PloS one. 2013;8(2):e56876 10.1371/journal.pone.0056876 23468887 PMC3585411

[7] Evans ND, Dimelow R, Yates J. Modelling of tumour growth and cytotoxic effect of docetaxel in xenografts. Computer methods and programs in biomedicine. 2014;114(1):e3–e13. 23948442 10.1016/j.cmpb.2013.06.014

[8] Simeoni M, Magni P, Cammia C, De Nicolao G, Croci V, Pesenti E, et al Predictive pharmacokinetic-pharmacodynamic modeling of tumor growth kinetics in xenograft models after administration of anticancer agents. Cancer research. 2004;64(3):1094–101. 14871843 10.1158/0008-5472.can-03-2524

[9] Yankeelov TE, Atuegwu N, Hormuth D, Weis JA, Barnes SL, Miga MI, et al Clinically relevant modeling of tumor growth and treatment response. Science translational medicine. 2013;5(187):187ps9–ps9. 10.1126/scitranslmed.3005686 23720579 PMC3938952

[10] Wang Z, Butner JD, Kerketta R, Cristini V, Deisboeck TS, editors. Simulating cancer growth with multiscale agent-based modeling Seminars in cancer biology; 2014: Elsevier. 10.1016/j.semcancer.2014.04.001 PMC4216775 24793698

[11] Macklin P, McDougall S, Anderson AR, Chaplain MA, Cristini V, Lowengrub J. Multiscale modelling and nonlinear simulation of vascular tumour growth. Journal of mathematical biology. 2009;58(4–5):765–98. 10.1007/s00285-008-0216-9 18781303 PMC3037282

[12] Quail DF, Joyce JA. Microenvironmental regulation of tumor progression and metastasis. Nature medicine. 2013;19(11):1423–37. 10.1038/nm.3394 24202395 PMC3954707

[13] Wu A, Liao D, Tlsty TD, Sturm JC, Austin RH. Game theory in the death galaxy: interaction of cancer and stromal cells in tumour microenvironment. Interface Focus. 2014;4(4):20140028 10.1098/rsfs.2014.0028 25097749 PMC4071511

[14] Delgado San Martin JA, Hare JI, Yates JWT, Barry ST. Tumour stromal morphology impacts nanomedicine cytotoxicity in patient-derived xenografts. Nanomedicine: Nanotechnology, Biology, and Medicine. 2015; 11(5): 1247–1252. 10.1016/j.nano.2015.02.007 25752857

[15] Smith NR, Baker D, Farren M, Pommier A, Swann R, Wang X, et al Tumor stromal architecture can define the intrinsic tumor response to VEGF-targeted therapy. Clin Cancer Res. 2013;19(24):6943–56. 10.1158/1078-0432.CCR-13-1637 24030704

[16] Augustin H. Translating angiogenesis research into the clinic: the challenges ahead. 2014. 10.1259/bjr/68078705 15456709

[17] Sengupta S. Clinical translational challenges in nanomedicine. MRS Bulletin. 2014;39(03):259–64.

[18] Prabhakar U, Maeda H, Jain RK, Sevick-Muraca EM, Zamboni W, Farokhzad OC, et al Challenges and key considerations of the enhanced permeability and retention effect for nanomedicine drug delivery in oncology. Cancer research. 2013;73(8):2412–7. 10.1158/0008-5472.CAN-12-4561 23423979 PMC3916009

[19] Nelander S, Wang W, Nilsson B, She QB, Pratilas C, Rosen N, et al Models from experiments: combinatorial drug perturbations of cancer cells. Molecular systems biology. 2008;4(1). 10.1038/msb.2008.53 PMC2564730 18766176

[20] Chen J, Layton AT, Edwards A. A mathematical model of O2 transport in the rat outer medulla. I. Model formulation and baseline results. American Journal of Physiology-Renal Physiology. 2009;297(2):F517–F36. 10.1152/ajprenal.90496.2008 19403646 PMC2724254

[21] Tyson JJ, Baumann WT, Chen C, Verdugo A, Tavassoly I, Wang Y, et al Dynamic modelling of oestrogen signalling and cell fate in breast cancer cells. Nature Reviews Cancer. 2011;11(7):523–32. 10.1038/nrc3081 21677677 PMC3294292

[22] Shackney SE, Shankey TV. Cell cycle models for molecular biology and molecular oncology: exploring new dimensions. Cytometry. 1999;35(2):97–116. 10554165 10.1002/(sici)1097-0320(19990201)35:2<97::aid-cyto1>3.3.co;2-x

[23] Tindall M, Please C, Peddie M. Modelling the formation of necrotic regions in avascular tumours. Mathematical biosciences. 2008;211(1):34–55. 18082225 10.1016/j.mbs.2007.09.002

[24] Frieboes HB, Smith BR, Chuang Y-L, Ito K, Roettgers AM, Gambhir SS, et al An integrated computational/experimental model of lymphoma growth. PLoS computational biology. 2013;9(3):e1003008 10.1371/journal.pcbi.1003008 23555235 PMC3610621

[25] Evans ND, Dimelow R, Yates J, editors. Modelling of tumour growth and cytotoxic effect of taxotere in xenografts. Biological and Medical Systems; 2012.

[26] Vaupel P, Schlenger K, Knoop C, Höckel M. Oxygenation of human tumors: evaluation of tissue oxygen distribution in breast cancers by computerized O2 tension measurements. Cancer research. 1991;51(12):3316–22. 2040005

[27] Evans ND, Chapman MJ, Chappell MJ, Godfrey K. Identifiability of uncontrolled nonlinear rational systems. Automatica. 2002;38(10):1799–805.

[28] Horoszewicz JS, Leong SS, Kawinski E, Karr JP, Rosenthal H, Chu TM, et al LNCaP model of human prostatic carcinoma. Cancer Res. 1983;43(4):1809–18. 6831420

[29] Usuda K, Saito Y, Sagawa M, Sato M, Kanma K, Takahashi S, et al Tumor doubling time and prognostic assessment of patients with primary lung cancer. Cancer. 1994;74(8):2239–44. 7922975 10.1002/1097-0142(19941015)74:8<2239::aid-cncr2820740806>3.0.co;2-p

[30] Popel AS. Theory of oxygen transport to tissue. Critical reviews in biomedical engineering. 1988;17(3):257–321. PMC5445261 2673661

[31] Hoffman RM. Orthotopic metastatic mouse models for anticancer drug discovery and evaluation: a bridge to the clinic. Investigational new drugs. 1999;17(4):343–60. 10759402 10.1023/a:1006326203858

[32] Fatt I, Giasson CJ, Mueller TD. Non-steady-state diffusion in a multilayered tissue initiated by manipulation of chemical activity at the boundaries. Biophysical journal. 1998;74(1):475–86. 9449348 10.1016/S0006-3495(98)77805-1 PMC1299400

[33] Harvitt DM, Bonanno JA. Re-evaluation of the oxygen diffusion model for predicting minimum contact lens Dk/t values needed to avoid corneal anoxia. Optometry & Vision Science. 1999;76(10):712–9. 10524787 10.1097/00006324-199910000-00023

[34] Fatt I, Shantinath K. Flow conductivity of retina and its role in retinal adhesion. Experimental eye research. 1971;12(2):218–26. 5119356 10.1016/0014-4835(71)90094-7

[35] Baish JW, Stylianopoulos T, Lanning RM, Kamoun WS, Fukumura D, Munn LL, et al Scaling rules for diffusive drug delivery in tumor and normal tissues. Proceedings of the National Academy of Sciences. 2011;108(5):1799–803. 10.1073/pnas.1018154108 PMC3033252 21224417

[36] Vaupel P, Kallinowski F, Okunieff P. Blood flow, oxygen and nutrient supply, and metabolic microenvironment of human tumors: a review. Cancer research. 1989;49(23):6449–65. 2684393

[37] Brahimi-Horn MC, Chiche J, Pouysségur J. Hypoxia and cancer. Journal of molecular medicine. 2007;85(12):1301–7. 18026916 10.1007/s00109-007-0281-3

[38] Gatenby RA, Gillies RJ. Why do cancers have high aerobic glycolysis? Nature Reviews Cancer. 2004;4(11):891–9. 15516961 10.1038/nrc1478

[39] Hsu PP, Sabatini DM. Cancer cell metabolism: Warburg and beyond. Cell. 2008;134(5):703–7. 10.1016/j.cell.2008.08.021 18775299

[40] Hanahan D, Weinberg RA. The hallmarks of cancer. Cell. 2000;100(1):57–70. 10647931 10.1016/s0092-8674(00)81683-9

[41] Lyng H, Sundfør K, Rofstad EK. Oxygen tension in human tumours measured with polarographic needle electrodes and its relationship to vascular density, necrosis and hypoxia. Radiotherapy and oncology. 1997;44(2):163–9. 9288845 10.1016/s0167-8140(97)01920-8

[42] Kirouac D, Onsum M. Using network biology to bridge pharmacokinetics and pharmacodynamics in oncology. CPT: pharmacometrics & systems pharmacology. 2013;2(9):e71. 24005988 10.1038/psp.2013.38 PMC4026631

[43] Ioslovich I, Gutman P-O, Seginer I. Dominant parameter selection in the marginally identifiable case. Mathematics and Computers in Simulation. 2004;65(1):127–36.

[44] Dubois L, Landuyt W, Haustermans K, Dupont P, Bormans G, Vermaelen P, et al Evaluation of hypoxia in an experimental rat tumour model by [(18)F]fluoromisonidazole PET and immunohistochemistry. Br J Cancer. 2004;91(11):1947–54. 15520822 10.1038/sj.bjc.6602219 PMC2409764

[45] Vermeulen PB, Colpaert C, Salgado R, Royers R, Hellemans H, Van den Heuvel E, et al Liver metastases from colorectal adenocarcinomas grow in three patterns with different angiogenesis and desmoplasia. The Journal of pathology. 2001;195(3):336–42. 11673831 10.1002/path.966

[46] Hanahan D, Weinberg RA. Hallmarks of cancer: the next generation. Cell. 2011;144(5):646–74. 10.1016/j.cell.2011.02.013 21376230

[47] Delgado San Martin J, Worthington P, Yates J. Non-invasive 3D time-of-flight imaging technique for tumour volume assessment in subcutaneous models. Laboratory Animals. 2014:0023677214562653. 10.1177/0023677214562653 25480658

[48] Pohjanpalo H. System identifiability based on the power series expansion of the solution. Mathematical biosciences. 1978;41(1):21–33.

[49] López C, Diana C, Barz T, Peñuela M, Villegas A, Ochoa S, et al Model-based identifiable parameter determination applied to a simultaneous saccharification and fermentation process model for bio-ethanol production. Biotechnology progress. 2013;29(4):1064–82. 10.1002/btpr.1753 23749438

