# Supplementary material for: Oxygen-Driven Tumour Growth Model: A Pathology-Relevant Mathematical Approach
Source: PLoS Comput Biol. 2015 Oct 30;11(10):e1004550. doi: 10.1371/journal.pcbi.1004550 (PMC4627780; doi:10.1371/journal.pcbi.1004550)
Supplement: S1 Text — (PDF) [file pcbi.1004550.s001.pdf]

Appendix

S1. ODM history description

S.1.1 Initial ODM

Our motivation comes from previous mathematical models based on hypoxia and concretely a model developed by Evans et al. [7], in which the compartments adopt a pseudophysical position in space. In general, our approach is a compilation of pre-existing modified models.

First, based on the Evans model we developed a semi-compartmental model. The dynamics of the initial model are based mainly on the mass action law. The key aspect of our models is the numerical identifiability and oxygen distribution.

Division rates are modelled through oxygen concentration, as a key component for energy production through oxidative phosphorylation. Oxidative phosphorylation is the main mechanism of energy production by cancer cells, 18 times more productive than glycolysis. The model also contains information of the outer proliferating layers (stem and partly differentiated cells undergoing division) characterised as a number of concentric proliferating compartments (N_P) with constant oxygen drop (see structural identifiability).

Hypoxic cells (cells experiencing scarce nutrient and oxygen delivery and quiescent, “dormant” cells) are also considered in the model as they play a very important role in intrinsic signalling for survival, proliferation and migration. Under hypoxic conditions (under 2mmHg), the cell reacts to the stress by producing several angiogenic and stress factors (HIF1α, VEGF, etc) which are responsible for the tortuous vascularisation of solid tumours.

Under extreme exposure to starvation and low oxygen levels, cells undergo necrosis which is typically localised in the core of the tumour. Necrotic death is hereby described in our model by the death rate (μ_q). These necrotic cells may be engulfed by the surrounding tissue, although to our knowledge, there has been no evidence reported in the literature. Further, although necrotic tissue may trigger an inflammatory response with subsequent macrophage recruitment, such a response has rarely been observed in immunocompromised animal models (cell components should migrate across the endothelium, see [s1, 2]). In this model, necrotic cells are not removed by the immune system and contribute to the tumour volume.

On the other hand, highly proliferating cells undergo numerous intrinsic processes involving mutations and DNA damage, which may activate the mitochondrial apoptotic pathway [s3]. However, the actual fate of the apoptotic cells is still unclear. They could remain, be reabsorbed or transported to other parts of the organism [s4]. In our model, the parameter (μ_p) represents the *apoptotic rate of cells*, which were not depleted by phagocytosis from the host immune system.

In our model, the communication between cell cycle and quiescent-hypoxic state is represented by a reversible reaction, where “deactivation”, (r_o) and further “reactivation”, (r_1) are reciprocally possible. This allows future implementation of synergic therapeutic combinations, namely radio-chemotherapy [s5].

Bringing all of these points together means the time rate of change of the proliferating volume is given by state equation (S1) whilst that of the quiescent region is state equation (S2) and necrosis is state equation (S3). Fig A shows a graphical representation of the model. Note that the ”reactivated” quiescent cells are evenly distributed across all the proliferating layers, thus N_P appears on equation S1.

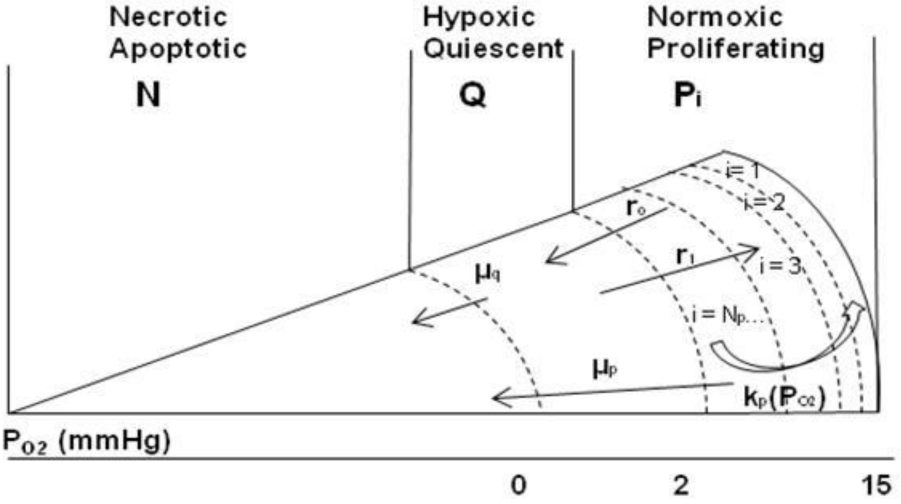

Figure A: radial representation of the semi-compartmental ODM

$$\frac{dV_{Pi}}{dt} = \left(k_P(P_{O2j}) - r_o - \mu_p\right)V_{Pj} + r_1 V_Q / N_P \tag{S1}$$

$$\frac{dV_Q}{dt} = r_o \sum V_{Pi} - (r_1 + \mu_q)V_Q \tag{S2}$$

$$\frac{dV_N}{dt} = \mu_p \sum V_{Pi} + \mu_q V_Q \tag*{(S3]}$$

The parameters of the model for tumour growth description are therefore $\theta =$

$[k'_P \; \mu_p \; \mu_q \; r_o \; r_1 \; k_R \;]$.

Disclaimer: Two different indexes have been defined here:

- i: refers to the natural counter of the shells
- j: refers to the index of the re-discretised layers.

In this model, the layers lose their integrity in every iteration, so P, works as a single compartment, which will be subsequently reorganised in different layers. So the interchange succeeds between 2 compartments P and Q effectively.

Knowing the algebraic equation describing oxygen partial pressure (eq. 6 of the main paper), the model described in eq. (S1) can transform into,

$$\frac{dV_{Pj}}{dt} = \Bigg(k_P{}'' \Bigg(P_{O2}^{max\,\prime} - (j-1)e^{-k_R'\cdot\left(\sqrt[3]{\sum V_k}-\sqrt[3]{V_Q}\right)}\Bigg(\Big(1+e^{2k_R'\cdot\sqrt[3]{\sum V_k}}\Big) - \Big(1+e^{k_R'\cdot\left(2\sqrt[3]{\sum V_k}-\sqrt[3]{V_Q}\right)}\Big)\Bigg)\Bigg) - r_o - \mu_p\Bigg)V_{Pj} + r_1 V_Q/N_P \tag{S4}$$

where in this case the proliferating constant has been regrouped as $k_P{}'' = k_P{}' \cdot C/N_P$ with units h^{-1}. Inversely the oxygen levels in the surrounding tissue have become apparent $P_{O2}^{max\,\prime} = P_{O2}^{max\,\prime} \cdot \frac{N_P}{C}$. A new term has also been added, substituting the whole tumour volume by the sum of its internal terms as follows: $V_T = \sum_{k=1}^{Np+2} V_k = \sum_{i=1}^{Np} V_{Pi} + V_Q + V_N$. The apparent oxygen uptake rate now has adopted the form: $k_R' = \sqrt{k_R/D} \cdot \sqrt[3]{\frac{4}{3}\pi}$.

The ODM model presented here is more flexible and provides with better results than the ODM presented in the main paper, but the relatively large number of parameters, makes the system not practically identifiable. However, in certain circumstances, this model would be more useful than the one presented in the main text of the paper.

S1.2 Advanced ODM

However, this rather complicated formulation can be simplified if we assume that hypoxia occurs at “a state of low cellular oxygen”. Since we calculate oxygen tension spatially, we can set up a “threshold” at which hypoxia occurs (in practical terms this is the oxygen levels at which hypoxia markers start to be expressed). We defined this “switch” as a smooth sigmoidal function. If we extend this definition to necrosis we can simulate the 1D maps of oxygen and hypoxia/necrosis (Fig. B / panels A-B). We also represented the switches of necrosis and hypoxia in the different layers, showing that the outer tumour layers present a lower proportion of hypoxia and necrosis.

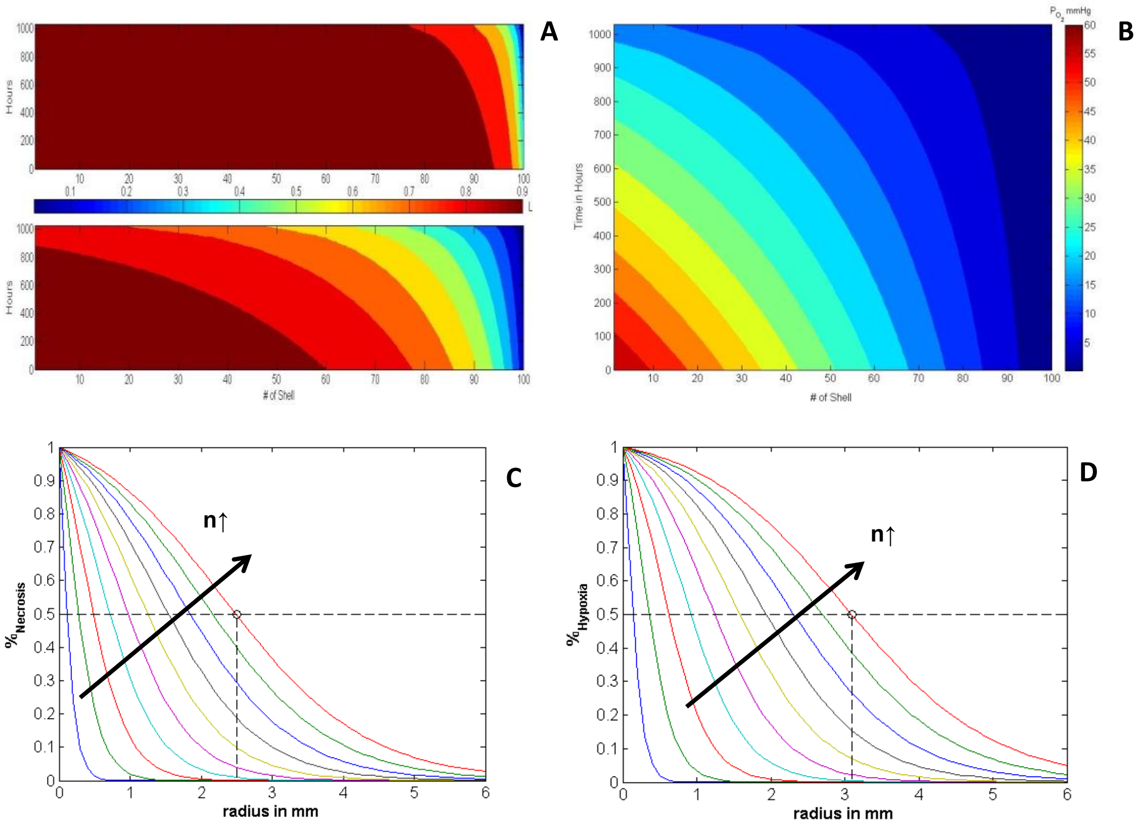

Figure B: Oxygen distribution and necrotic/hypoxic switches. (A) (Top) time-spatial distribution of hypoxia, (Bottom) of necrosis. (B) time-spatial distribution of oxygen. (c-d) quality of sigmoidal switches for an increasing number of shells (n=2-20).

Following, the version of the ODM presented in section S1.2 is just an extended version of the discretisation throughout the whole tumour combined with the sigmoid switches for hypoxia and necrosis, resulting in the model presented in the main paper.

S2. Assumptions of the ODM

We revisit here the main assumptions of the model presented in the main paper. This time we want to explore the mathematical spaces of the assumptions and ground our decisions.

S2.1 Oxygen Diffusion

We will first describe part of the rationale of oxygen diffusion in the model. There are two possible kinds of hypoxia characterisation: acute (perfusion related) or chronic (diffusion related). It is important to note that, different markers of hypoxia might be looking at different kind of hypoxia [s6], which suggests the need to analyse the problems on a case specific basis.

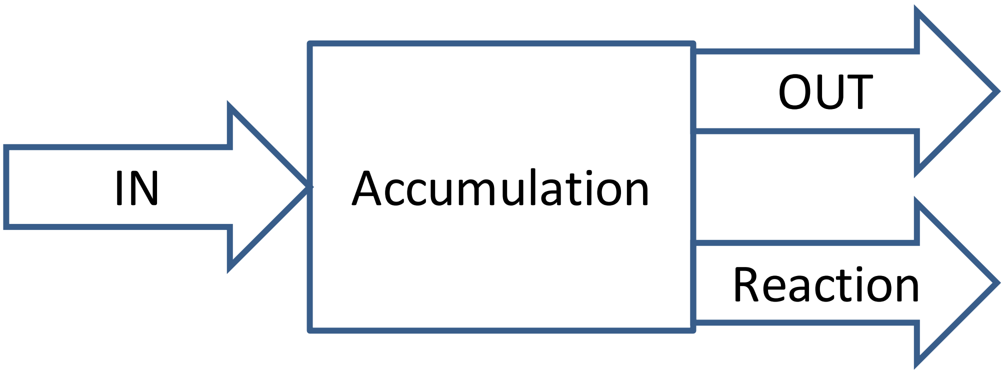

Figure C: Diagram describing mass conservation within the tumour

Oxygen diffusion is described by means of molecular transport equations. For any system we can define the mass conservation law as $IN - OUT = Accumulation - Reaction$, as depicted in Fig CFigure , following the Stokes mass conservation (S5.

$$J_{IN} - J_{OUT} = \nabla J = \frac{\partial \phi}{\partial t} - R \quad \text{(S5)}$$

with J as matter flux, ϕ as concentration, t time and R reaction. Let us define Fick's first Law, which states that matter flux is proportional to the change in concentration such that $J = -\mathrm{D}\nabla\phi$, with D as diffusivity (equation (S6). In general engineering text books

this can be found as $FLOW = \frac{POTENTIAL}{RESISTANCE}$, which holds for electrostatics, heat equations and momentum conservation.

$$\nabla(-\mathrm{D}\nabla\phi) = \frac{\partial \phi}{\partial t} - R \tag{S6}$$

Let us define concentration as oxygen partial pressure $P_{O2} = \phi$ and consider the quasi-steady state approximation $\frac{\partial P_{O2}}{\partial t} = 0$. If diffusion is independent of position, $\mathrm{D} \neq \mathrm{f(r)}$ we derive the eq. (S7).

$$\frac{\partial P_{O2}}{\partial t} = -\nabla \cdot (D(r) \cdot \nabla P_{O2}) + R_{O2} \xrightarrow{\frac{SS}{D} \neq f(r)} 0 \approx -D \cdot \nabla^2 P_{O2} + R_{O2}(P_{O2}) \tag{S7}$$

Above, D, P_{O2} and R_{O2} are respectively the diffusion coefficient, oxygen partial pressure and oxygen uptake rate by the cells. Rearranging the equation and assuming spherical symmetry, we obtain the equation 10 of the main paper.

S2.2 Limited Oxygen Distribution

Having introduced the oxygen diffusion problem we proceed with the ODM specific assumptions described by Fig D.

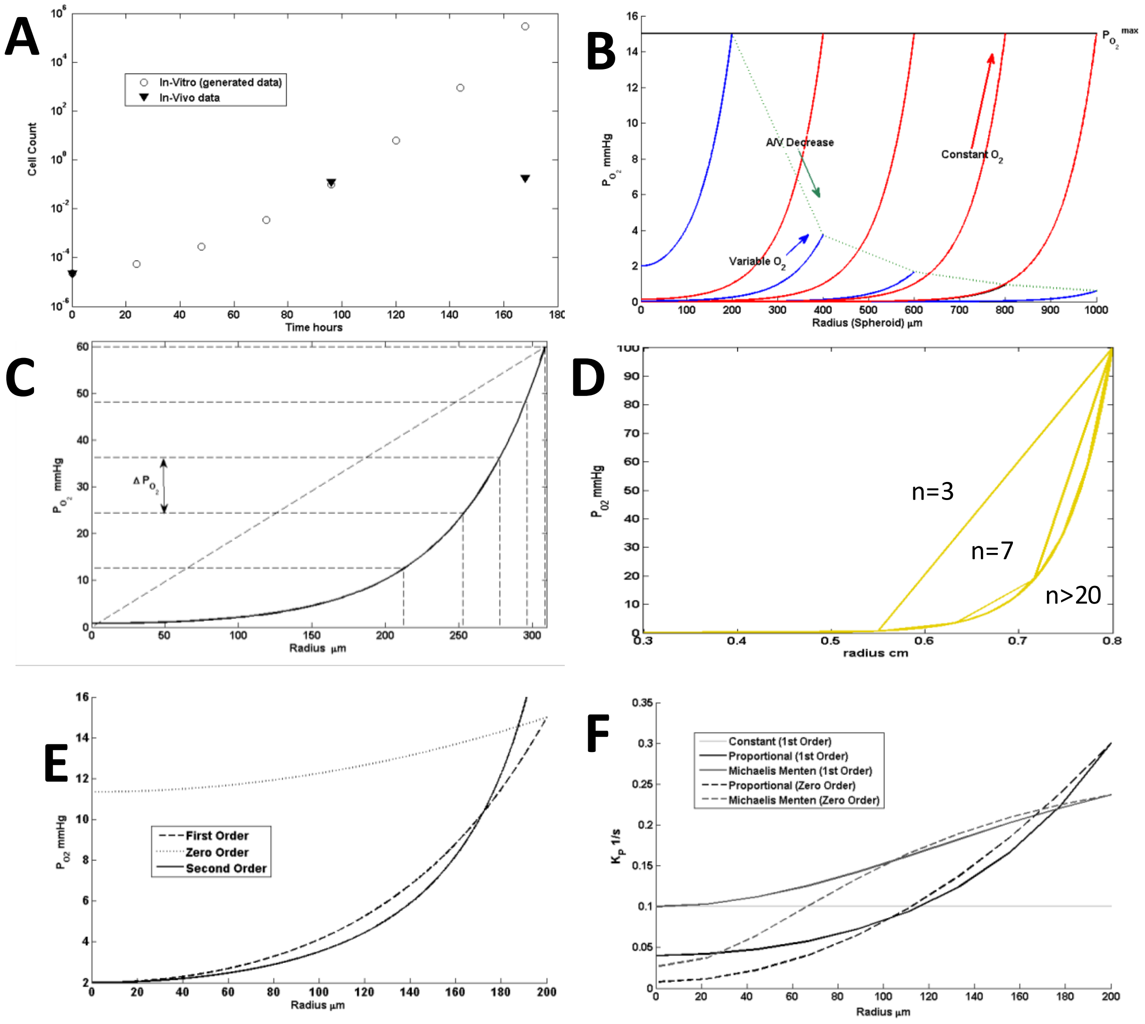

Figure D: (A) Experimental and generated data of in-vivo and in-vitro growth curves of the A431 cell line. (B) Geometric assumption: surface area/volume ratio decreases when the volume increases. This affects oxygen distribution. (C) Rediscretisation. The radial points are not chosen equidistantly, but at the point at which the drop in oxygen is constant. This linearises the equations. (D) Optimal number of shells. (E) Model structure for the oxygen uptake rate, assuming 0, 1st and 2nd order. (F) Test of general tumour growth model structure, with constant, proportional and Michaelis-Menten structures.

Some cell lines grow faster in-vitro than in-vivo, but it is not until a certain point, that this difference starts to create a quantitative margin. We simulated the in-vitro exponential growth (with a doubling time of ~18h) of the A431 cell line and plotted it along with in

vivo data. We see after the first 4 days, the growth in vivo and in vitro are equivalent as shown in Fig D. However, somewhere between day 4 and 7 there is an inflection point, which in the literature is often referred to as “the angiogenic switch”. In our opinion, this is the switch from a cell suspension to a real organ, where oxygen delivery occurs from diffusion limited blood vessels rather than directly from the medium. This involves, not only development of a tumour vasculature, but also recruitment of stromal cells creating a favourable microenvironment.

In a xenograft, the tumour is only in contact with the well vascularised epithelial tissue of the lower ectodermic layers from one side and the body wall from the other side. As opposed to the vascularised somatic tissue, the angiogenic vasculature of the tumour may not carry blood cells, and often transports low oxygen/nutrient plasma. Therefore, we can consider the effective access to blood carrying vessels is corrected through a shrinking lateral area-volume ratio (in other words, access to oxygen decreases with tumour growth). In the clinic, the situation may be reversed, thus the vasculature may be mature and tissue invasiveness may lead to the opposite trend (access to oxygen increases with tumour development).

The heuristics applied here were also implemented in some examples in the literature [s7]. Fig D / panel B elucidates the comparison between the constant oxygen supply from the peripheral capillaries at 60 mmHg as epithelial tissue from the body wall and a variation with the V/A ratio as an exponential 3/2 function represented by eqs. (S8)-(S9).

Constant Supply	$$P_{O2}^{max} = 60mmHg$$	(S8)
Shrinking Supply	$$P_{O2}^{max} = 60 \cdot \left({}^{V_{To}}/_{V_T} \right)^{2/3} \ mmHg$$	(S9)

Above, V_{To} and V_T are the initial and final volumes of tumour respectively. The "shrinking supply" phenomenon has been vastly reported in the literature.

S2.3 Spatial Discretisation

In this model, as will be shown later in this section, the discretisation plays two main roles. One is the reduction of the total error associated with it and the other is transforming the model into a structurally identifiable model.

Hereby, there are 3 possibilities to discretise the model dividing evenly:

- Volume: thickness would be uneven and proportional to its mean oxygen concentration.
- Radius: Error still increases with the gradient of P_{O2}
- Oxygen Partial Pressure Change: constant error function.

In this sense, we applied the last and more consistent method as shown in Fig D / panel C. The plot shows the division of 5 intervals according to the even oxygen decay.

To explore the minimum number of shells needed to obtain an acceptable accuracy, we explored the surface of oxygen diffusion. Results show that **n=20** provides a sufficient accuracy (see Fig. D / panel D), at a tolerance of 0.3% in volume.

S2.4 Oxygen uptake rate

Oxygenation in tumours is often lower than in other organs (up to 6 times less [s8]). Furthermore, hypoxic cells have little access to oxygen, for which metabolism slows down and to a pseudo-dormant state. In this first assumption, we consider 3 possible forms of the oxygen uptake rate, described by eqs. (S10)-(S12).

Zero Order	$R_{O2} = k_R' \cdot P_{O2}{}^{0}$	(S10)
First Order	$R_{O2} = k_R' \cdot P_{O2}{}^{1}$	(S11)
Second Order	$R_{O2} = k_R' \cdot P_{O2}{}^{2}$	(S12)

Solving the problem described in eq. 6 of the main text with a given value of k_R'= 2 cm^{-1} combined with the different expressions of the uptake rate described in eqs. (S10(S12), we obtain oxygen curves as described in Fig D / panel E. The curve for first order is similar to a double exponential, which can be further represented by a single exponential and an error function of the form: $P_{O2} = C \cdot e^{k_R' t} + \varphi$, where $\varphi \epsilon \Omega \epsilon \mathbb{R}$ is an error function. In other words, the zero order expression plus an error function would give you the first order expression. In this case, we have no previous information to calculate the error function. Although the second order expression yields a similar oxygen distribution, it cannot be solved analytically. Also, the oxygen uptake has been reported to follow first order dynamics numerous times. These arguments drove us to the choice of the first order expression by eq. (S11).

S2.5 Proliferation constant as function of the Oxygen Diffusion

The proliferation constant should be oxygen dependent in our models (k_p (P_{O2})). We propose here three alternatives considering constant, proportional and Michaelis-Menten (MM) like types of equations.

Let k_p (P_{O2}) describe the oxygen dependent proliferation function and k_p' the proliferation constant as a numeric parameter of the system, then we postulate the equations (S13)-(S15).

Constant
$$k_P = k_P' {P_{O_2}}^0 \quad \text{(S13)}$$

Proportional
$$k_P = k_P' \cdot P_{O_2} \quad \text{(S14)}$$

Michaelis-Menten (MM)
$$k_P = k_P' \cdot \frac{P_{O_2}}{K_M + P_{O_2}} \quad \text{(S15)}$$

These equations yield the results in Fig D / panel F, where the proliferating rate is plotted against the tumour radius. It can be observed that there is a saturation characteristic of the MM model, which both yield very similar results for lower levels. In the range of the observed oxygen diffusion (maximal 200 μm) the values of both functions, Proportional and MM are very similar. Both can yield good results to explain growth retardation. In our model we used eq. (S14) for simplicity.

S3. Indentifiability problem

Parameter identifiability has been defined as: “Given a model of the system and specific input-output experiments, we ask, if the data were error free, could the parameters of the model be uniquely determined?” [36]. The concept of identifiability is directly linked to the model inference. In other words, we want to know whether our estimations really represent the model reality.

Two different kinds of numerical identifiability are commonly handled in the literature: structural and practical. We analysed our model from the perspective of both identifiability tests, prioritising the statistical confidence of our model inference.

S3.1 Structural Identifiability

As the name indicates, the structural identifiability depends on the intrinsic structure of the model and was first introduced in compartmental models by Bergman and Astrom [s9]. In linear systems the availability of methods is extensive, though in non-linear systems the number of methods remains reduced. Chappell et al. [s10] discussed some methods which have been widely applied in biological systems.

Here we applied the Taylor’s series problem to our observables (eq. 11 in main paper). First we identify our observable (tumour volume) at time t:

$$V_T(t,\theta) = \sum V_i(t,\theta). \quad \textbf{(S16)}$$

Let us approximate V_T using a Taylor series expanded around the initial tumour volume. For simplicity we chose evenly distributed initial volumes across the tumour $V_i(0,\theta)$ = V_{To}/n. The Taylor coefficients result in:

$$\text{0 order: } f_0 = \sum V_i(t,\theta) = V_{To}; \quad \textbf{(S17)}$$

1 order $f_1 = \sum \frac{dV_i(t,\theta)}{dt} = k_p \left(1 - \frac{n+1}{2\cosh(-k_R{}'\sqrt[3]{V_{To}})}\right);$ **(S18)**

2 order $f_2 = \sum \frac{d^2V_i(t,\theta)}{dt^2} = k_p \cdot f_1 \cdot \left(1 - \frac{n+1}{2\cosh(-k_R{}'\sqrt[3]{V_{To}})} \cdot \left[1 + V_{To}{}^{1/3} \cdot\right.\right.$ **(S19)**

$\left.\left.k_R{}' \cdot \tanh(-k_R{}'\sqrt[3]{V_{To}})\right]\right).$

For simplicity and due to the fact that the constants do not play any role in the structural identifiability, we left lumped constants together. According to Taylor's theorem each coefficient is linearly independent from the rest and therefore uniquely identifiable. First and second coefficients (eqs. (S18) and (S19) show 2 linearily independent equations with two parameters. If we rearrange and combine the two equations in terms of k_R', we obtain:

$$f_2 = f_1 \cdot \left(1 + \frac{2V_{To}{}^{1/3} \cdot k_R{}' \cdot \sinh(-k_R{}'\sqrt[3]{V_{To}})}{2\cosh\left(-k_R^{'}\sqrt[3]{V_{To}}\right) - (n+1)}\right) \quad \textbf{(S20)}$$

To solve equation (S20), we face a similar challenge to solving equations of type: $y = x \cdot e^x$. If the domain is real and positive volumes: $\forall t, \theta; V_{To} > 0 \in \mathbb{R}$ and also the parameter values $\theta > 0 \in \mathbb{R}$, there is a unique solution confined in our physically feasible ranges. This makes the problem **locally identifiable**, which is a sufficient condition. However, special care has to be taken in the practical identifiability and significance of the parameters.

S3.2 Practical Identifiability

We refer to practical identifiability of nonlinear systems to the data-sensitive unique parameter estimations. There are a number of methods described in the literature, although here we will use the sensitivity data based on analytical Fisher Information Matrix (FIM) and sensitivity matrix (S), as described by Refs. [s11] and [43], where the sensitivity matrix is a $r \cdot t \times j$ dimensional array with elements $S_{rxt,j} = \left.\frac{\partial V_r}{\partial \theta_j}\right]_t$ defined at each time (t), for each parameter j and for each measured output r (in this case tumour volume). The Fisher Information matrix was defined as $FIM = S^T \cdot S$, whereas, the covariance matrix (Co) has the form $Co = \frac{1}{FIM}$. Generally in the notation, we will refer the total parameter vector as $\theta_j \in \theta \in \mathbb{R}^n$. For non identifiable systems a subset of parameters based upon unidentifiable parameters can be selected, assuming a set of physiologically plausible parameters which are fixed. We used a rank revealing factorisation through singular value decomposition of the normalised sensitivity matrix $\hat{S} = U \cdot \Sigma \cdot V^T$, obtaining the rectangular diagonal matrix (Σ) of the nonnegative real numbers of the factorization. Further, the normalised sensitivity matrix ($\hat{S}$) adopts the form, $\hat{S} = S \cdot \tilde{\theta}/_{\tilde{Y}}$. Then we calculate the collinearity index (γ(S)) and the condition number (κ(S)), being $\kappa \quad = \frac{\sigma_1}{\sigma_{Last}}$ and $\gamma \quad = \frac{1}{\sigma_{Last}}$, where σ_1 and σ_{Last} are the first and last values of the factorised diagonal matrix (diagonal of Σ). Values for κ and γ higher than a fixed threshold 1000 and 10 respectively was considered unidentifiable. We then can determine the number of elements of Σ which fulfil the above condition, i.e. the number of "identifiable parameters" $n_{ip.}$ The remaining parameters are the "unidentifiable parameters" n_{up}, which in that case would be fixed.

S3.3 Standard errors

The standard errors (SE) of the ODM has been calculated by means of the "Fisher information matrix", FIM:

$$FIM = {S^{-1} \times S}/{\sigma_{Y-\bar{Y}}} \therefore SE = \sqrt{diag(FIM^{-1})}. \quad \textbf{(S21)}$$

Above $\sigma_{Y-\bar{Y}}$ represents the residual of the model estimation.

This method would provide some idea about the estimates, although it would be more accurately represented by bootstrapping or other resampling methods for better accuracy.

S4. Oxygen perfusion - observations

In this section we will report some key features related to oxygen diffusion observed in preclinical tumours and reported in the literature, supporting some of the assumptions made in the model.

Tumours are highly dynamic irregular complex structures of cells, but most importantly they are 3D structures. One interesting phenomenon to support this idea, as commented in the main text is that some necrotic areas originate in areas close to the vessels (CD31 positive, Fig E). This reminds us that immunohistochemical (IHC) data is data of a cross section of a 3D tumour, where vessels may be visibly occluded or apparently wide open but occluded somewhere else.

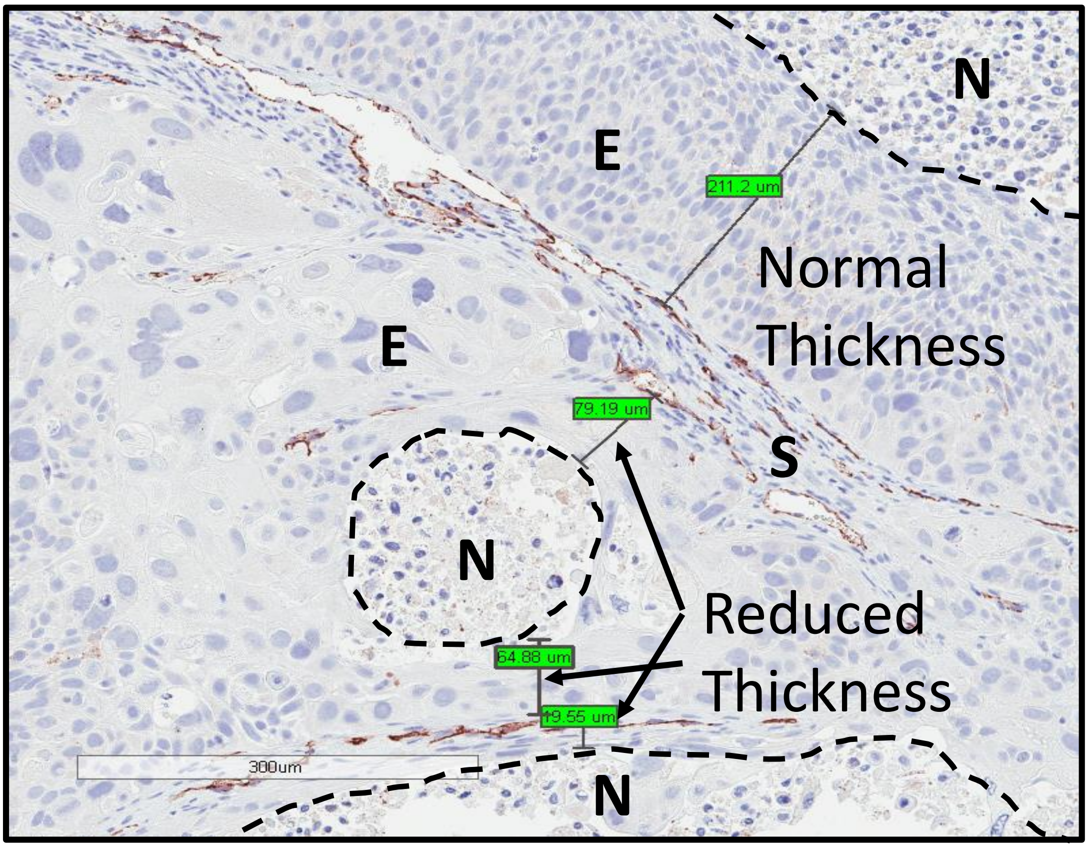

Figure E: IHC example of a lung explant tumour stained for CD31 and counterstained with Hematoxilin. The epithelial (E) thickness from the Stroma (S) to the necrotic (N) core ranges from around 200 µm, "Normal thickness", to 20-80µm "Reduced thickness."

Table S1 shows the oxygen reach reported by different authors and the calculation of the oxygen uptake rate coefficient according to the equation 12 in the main text. We took the

“oxygen reach” as a 2 mmHg threshold with external oxygen tension of 60mmHg. The output of this calculation provided us with values of k_R’ ~200-400 cm^{-1} (Table A).

Table A: Literature values of oxygen reach and consequent k_R’ values.

O_2 reach Distance (µm)	Source	k_R' (cm^{-1})
50-100	Baish 2001 [35]	876-438
40-200	Dewhirst 1994 [s12]	1095-218
100-200	Brahimi-Horn 2007 [37]	438-218
100-200	Carmeliet 2000 [s13]	438-218
150	Krohn 2008 [s14]	292
70	Krohn 2008 [s14]	626
200	Own observations	218

To elucidate the effect of vasculature in k_R’ we drew a cartoon to show that hypoxia appears at approximately 250-400 µm from non-vascularised tissue, triggering secretion of VEGF, PDGF and other angiogenesis promoters. This is well known as the “angiogenic switch”. Thereafter, as the tumour grows it develops its own vasculature which is more distributed and angiogenic the lower this parameter is (Fig F / panel A). Reduction of the oxygen uptake rate translates into an apparent longer oxygen penetration in the tissue (Fig F / panel B) and thus greater proliferation rates.

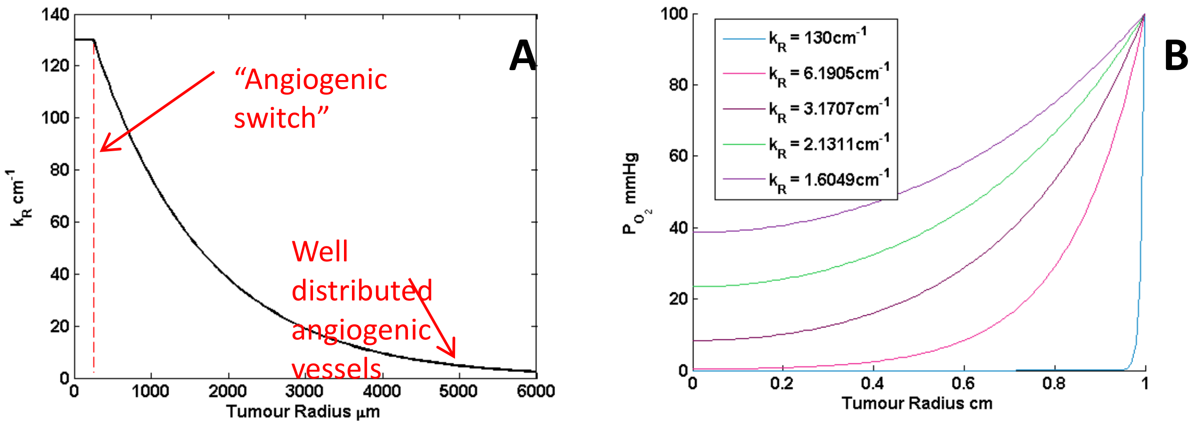

Figure F: Theoretical results for avascular oxygen uptake rate (A) Cartoon on the effect of the angiogenic switch in k_R’. (B) Example of k_R’ effect on oxygen penetration, calculated with Eq. (2).

S5. Raw results

The plots of the averages across data sets are provided on Fig G.

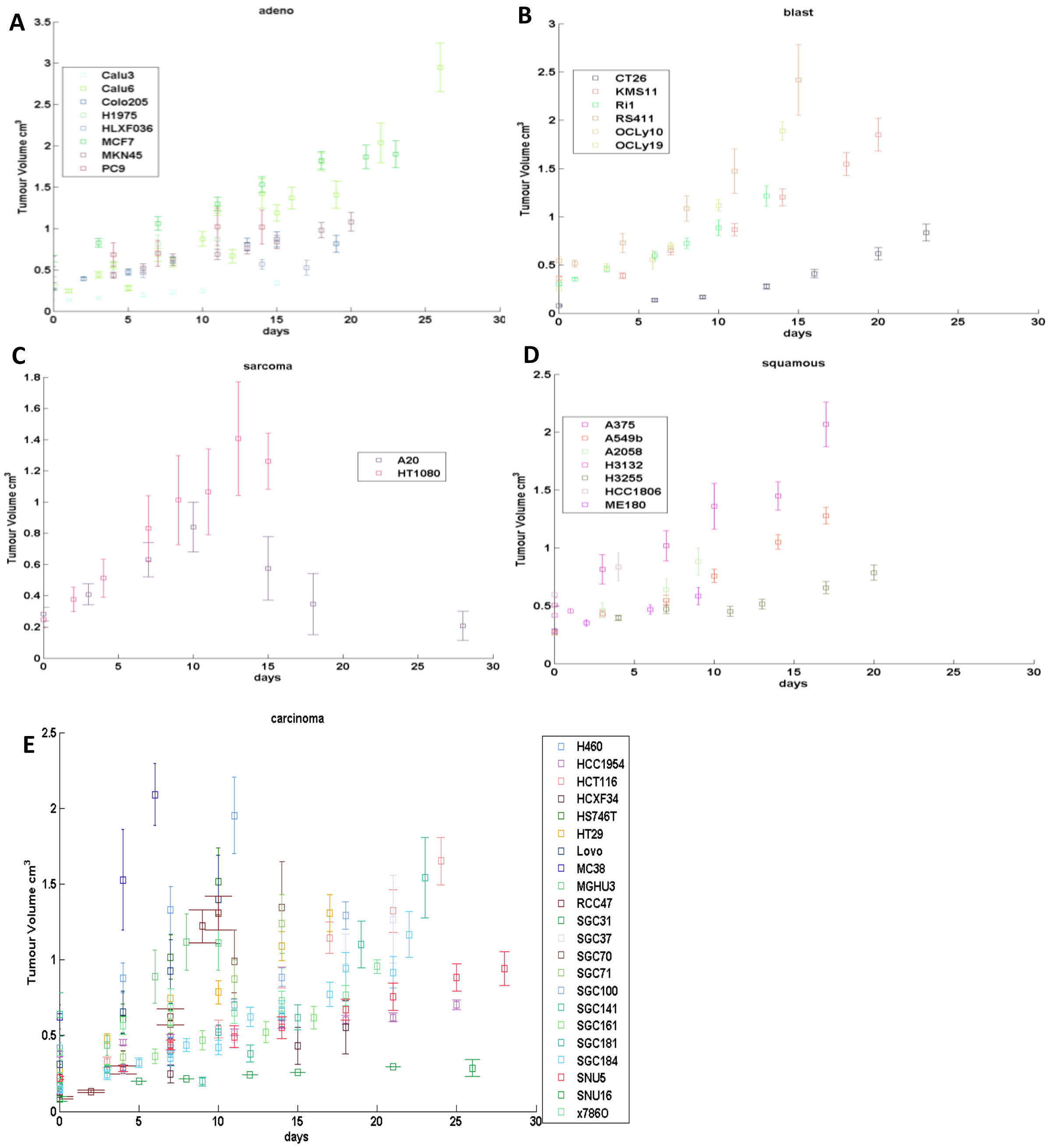

Figure G: data sets used in for the parameter fit ordered by the type of cancer. (A) are adenocarcinoma cell lines, (B) fibroblast-derived cell lines, (C) Sarcoma cell lines, (D) squamous carcinoma cell lines and (E) are the rest of carcinomas mostly pancreatic, kidney and gastroenteric cell lines.

S6. Glossary of Appendix

Symbol	Name	Units	Symbol	Name	Units
V_{Pi}	Proliferating tumour volume	cm^3	K_N	Necrosis constant	mmHg
V_i^H, V_Q	Hypoxic/ Quiescent volume	cm^3	r_o	Inactivation constant	day^{-1}
V_i^N, V_N	Necrotic volume	cm^3	r_1	Re-activation constant	day^{-1}
V_T	Tumour volume	cm^3	μ_p	Death constant (proliferation)	day^{-1}
V_{To}	Initial tumour volume	cm^3	μ_q	Death constant (quiescence)	day^{-1}
r_T	Tumour radius	cm	J	Generic flux	
i, j, k	Layer #	-	ϕ	Generic concentration	
n, N_p	# of Layers	-	R	Generic Reaction rate	
$P_{O_2,i}$	Oxygen Pressure	mmHg	FIM	Fisher Information Matrix	
$P_{O_2}^{max}$	Blood Oxygen Levels	mmHg	S	Sensitivity matrix	
t	Time	day	Σ	Covariance matrix	
k_p	Proliferation rate	$(day.mmHg)^{-1}$	γ	Collinearity index	

k_R'	Oxygen uptake rate	cm^{-1}	κ	Condition number
K_H	Hypoxia constant	mmHg	θ	Genericallly, parameters

S7. References

1. Liou P, Bader L, Wang A, Yamashiro D, Kandel JJ. Correlation of tumor-associated macrophages and clinicopathological factors in Wilms tumor. Vascular cell. 2013;5(1):5.
2. Sager HB, Middendorff R, Rauche K, Weil J, Lieb W, Schunkert H, et al. Temporal patterns of blood flow and nitric oxide synthase expression affect macrophage accumulation and proliferation during collateral growth. J Angiogenes Res. 2010;2:18.
3. Conradt B. Genetic control of programmed cell death during animal development. Annual review of genetics. 2009;43:493.
4. Kroemer G, Levine B. Autophagic cell death: the story of a misnomer. Nature reviews Molecular cell biology. 2008;9(12):1004-10.
5. Wakeling AE, Guy SP, Woodburn JR, Ashton SE, Curry BJ, Barker AJ, et al. ZD1839 (Iressa) an orally active inhibitor of epidermal growth factor signaling with potential for cancer therapy. Cancer research. 2002;62(20):5749-54.
6. Padhani AR, Krohn KA, Lewis JS, Alber M. Imaging oxygenation of human tumours. European radiology. 2007;17(4):861-72.
7. Lowengrub JS, Frieboes HB, Jin F, Chuang Y, Li X, Macklin P, et al. Nonlinear modelling of cancer: bridging the gap between cells and tumours. Nonlinearity. 2010;23(1):R1.
8. Vaupel P, Kallinowski F, Okunieff P. Blood flow, oxygen and nutrient supply, and metabolic microenvironment of human tumors: a review. Cancer research. 1989;49(23):6449-65.
9. Bellman R, Åström KJ. On structural identifiability. Mathematical Biosciences. 1970;7(3):329-39.
10. Chappell MJ, Godfrey KR, Vajda S. Global identifiability of the parameters of nonlinear systems with specified inputs: a comparison of methods. Mathematical Biosciences. 1990;102(1):41-73.
11. López C, Diana C, Barz T, Peñuela M, Villegas A, Ochoa S, et al. Model-based identifiable parameter determination applied to a simultaneous saccharification and fermentation process model for bio-ethanol production. Biotechnology progress. 2013;29(4):1064-82.
12. Dewhirst MW, Secomb TW, Ong ET, Hsu R, Gross JF. Determination of local oxygen consumption rates in tumors. Cancer research. 1994;54(13):3333-6.
13. Carmeliet P, Jain RK. Angiogenesis in cancer and other diseases. nature. 2000;407(6801):249-57.
14. Krohn KA, Link JM, Mason RP. Molecular imaging of hypoxia. Journal of Nuclear Medicine. 2008;49(Suppl 2):129S-48S.
